# Red Rice Seed Coat Targeting SPHK2 Ameliorated Alcoholic Liver Disease via Restored Intestinal Barrier and Improved Gut Microbiota in Mice

**DOI:** 10.3390/nu15194176

**Published:** 2023-09-27

**Authors:** Yuxu Chen, Zhiye Zhao, Shancheng Guo, Yaxian Li, Haiaolong Yin, Lei Tian, Guiguang Cheng, Ye Li

**Affiliations:** 1 Faculty of Food Science and Engineering, Kunming University of Science and Technology, Kunming 650500, China; 2 School of Basic Medicine, Kunming University of Science and Technology, Kunming 650500, China

**Keywords:** red rice seed coat, alcoholic liver disease, SPHK2, gut microbiota, intestinal barrier

## Abstract

Alcoholic liver disease (ALD), leading to the most common chronic liver diseases, is increasingly emerging as a global health problem, which is intensifying the need to develop novel treatments. Herein, our work aimed to estimate the therapeutic efficacy of red rice (*Oryza sativa* L.) seed coat on ALD and further uncover the underlying mechanisms. Red rice seed coat extract (RRA) was obtained with citric acid–ethanol and analyzed via a widely targeted components approach. The potential targets of RRA to ALD were predicted by bioinformatics analysis. Drunken behavior, histopathological examination, liver function, gut microbiota composition and intestinal barrier integrity were used to assess the effects of RRA (RRAH, 600 mg/kg·body weight; RRAL, 200 mg/kg·body weight) on ALD. Oxidative stress, inflammation, apoptosis associated factors and signaling pathways were measured by corresponding kits, Western blot and immunofluorescence staining. In ALD model mice, RRA treatment increased sphingosine kinase 2 (SPHK2) and sphingosine-1-phosphate (S1P) levels, improved gut microbiota composition, restored intestinal barrier, decreased lipopolysaccharide (LPS) levels in plasma and the liver, cut down Toll-like receptor 4 (TLR4)/Nuclear factor kappa B (NF-κB) pathways, alleviated liver pathological injury and oxidative stress, attenuated inflammation and apoptosis and enhanced liver function. To sum up, RRA targeting SPHK2 can ameliorate ALD by repairing intestinal barrier damage and reducing liver LPS level via the TLR4/NF-κB pathway and intestinal microbiota, revealing that red rice seed coat holds potential as a functional food for the prevention and treatment of ALD.

## 1. Introduction

ALD is a kind of typical liver disease in the world, which seriously endangers people’s health. It is an illness triggered by prolonged immoderate alcohol consumption that can progress to alcohol-associated liver injury, alcoholic hepatitis, alcoholic fibrosis, and finally alcoholic cirrhosis and even liver cancer [1,2]. In recent years, the incidence of ALD is rising, and it is becoming the second major cause of liver injury following viral hepatitis [3]. ALD is a primary outcome of the complex interaction among various factors, such as nutritional imbalance, enterogenic endotoxin, endoplasmic reticulum stress, inflammatory mediators and oxidative stress directly or indirectly induced by the ethanol metabolism and derivatives [4,5]. It is especially enterogenic endotoxemia caused by damaging the intestinal barrier, endotoxin activation and changes of the intestinal microbiota, that play a pivotal role in the occurrence and development of ALD [6]. The SPHK2-mediated signaling pathway presents a protective effect in ALD by regulating hepatic lipid metabolism [7], and SPHK2 is a rate-limiting enzyme of S1P formation, which is the key regulator of the sphingolipid signaling pathway [8]. S1P plays an important role in protecting the intestinal barrier [9]. D-LA is a marker of intestinal barrier integrity in intestinal microbiota disorder [10,11]. LPS significantly increases the expression of pro-inflammatory factors, which exacerbates inflammation via the NF-κB activation [12]. The NF-κB pathway is the central cell signaling pathway in the liver and gut that activities multiple stress-related and inflammatory mediators, which aggravates ALD progression and leads to the occurrence of liver fibrosis [13]. Although there are reports on natural products for the treatment of ALD [14], currently, the treatment of ALD is unsatisfactory and there is no effective drug for its treatment in clinics yet. The fatality rate caused by ALD is increasing year by year [15].

Red rice (*Oryza sativa* L.) is a grass rice close to wild rice (*Oryza*) and weed rice. It is classified as indica, associated with japonica rice, and it is known as red rice because of the brownish-red coat [16,17]. Red rice is delicious and nutritious, and the chemical compositions of it contain flavonoids, terpenoids, phenols, etc. [18,19]. The extract of red rice seed coat has various physiological effects such as eliminating free radicals, immunoregulation, improving stress resistance and delaying aging [20,21]. The antioxidant capacity of red rice was remarkably stronger than that of white rice, because catechin and epicatechin contents were substantial in red rice [22]. The red rice could inhibit dipeptidyl peptidase-4 expression and stimulate glucagon-like peptide 1 secretion in type Ⅱ diabetes [23]. However, the effects of red rice on ALD and the definite mechanism have not been reported.

In this work, the seed coat of red rice was extracted with citric acid–ethanol and analyzed by a widely targeted components approach, which mainly consisted of flavonoids, alkaloids, phenolic acids, lipids and amino acids and derivatives. RRA treatment evolved a variety of protective actions on ALD by alleviating oxidative stress, attenuating inflammation, and inhibiting apoptosis. Bioinformatics analysis and experiment validation showed that RRA treatment ameliorated ALD by upregulating SPHK2, restoring the intestinal barrier and improving gut microbiota in mice.

## 2. Materials and Methods

### 2.1. Acquisition and Analysis of ALD Differentially Expressed Genes (DEGs)

ALD and normal liver tissue mRNA expression profiling by array were amassed in the Gene Expression Omnibus (GEO) (http://www.ncbi.nlm.nih.gov/geo/ (accessed on 27 March 2023)) [24]. We collected 6 ALD Model and 6 Control groups from GSE86002. In order to discern the ALD DEGs, we applied R (version 4.3.1) packages dplyr and limma to analyze the GSE86002 dataset. R packages ggplot2 and cluster Profiler were used to carry out the DEGs Gene Ontology (GO) analysis.

### 2.2. The Potential Targets Analysis of RRA on ALD

The main compounds of red rice seed coat were chosen as the result of widely targeted components analysis. We retrieved and screened these connected targets of red rice seed coat compounds from Swiss Target Prediction (http://www.swisstargetprediction.ch/ (accessed on 5 September 2023)) [25]. Then, the ALD-associated genes were filtrated from DisGeNET (https://www.disgenet.org/ (accessed on 3 February 2021)) [26]. We screened overlapping genes associated with ALD, potential targets of red rice seed coat and DEGs of GSE86002 with corresponding R packages. The Kyoto Encyclopedia of Genes and Genomes (KEGG) was used to analyze the potential cellular signaling pathways associated with overlapping genes, and GO was used to visualize the molecular function, cellular component and biological process of overlapping genes. KEGG and GO visualized analysis were operated with the R program.

Protein interaction (PPI) of overlapping genes was ascertained by STRING (https://cn.string-db.org/ (accessed on 26 July 2023)) [27], and Cytoscape (3.8.0) was used to construct the interaction network. The RRA compounds and target networks of red rice seed coat against ALD were built with Cytoscape (3.8.0).

### 2.3. The Extract of Red Rice Seed Coat Preparation

Red rice was purchased from Mojiang, Yunnan Province, China. It was extracted according to the procedure mentioned in [28] with a little modification. In short, the powdered seed coat of red rice was filtered with an 80-mesh sieve and extracted 3 times with citric acid–ethanol by a sonication-assisted method, for 0.5 h each time in a 1:16 ratio of material/solution, 4500 rpm centrifugation for 10 min. Then, the extract was acquired via evaporation and vacuum lyophilized in the supernatant.

### 2.4. Widely Targeted Components Analysis of RRA

Samples were prepared according to the procedure outlined in [29]. RRA components were identified based on a public related database and local self-built MWDB databases (Metware Biotechnology Co., Ltd., Wuhan, Hubei, China). RRA component quantification was carried out in multiple reaction monitoring mode (MRM) using triple quadrupole mass (QQQ) spectrometry. The relative contents of RRA components were represented with characteristic mass spectra peak area integrals.

### 2.5. Animal Experiments

Male C57BL/6J mice, aged 8 weeks, were obtained from Beijing SPF Animal Technology Co., Ltd. (Beijing, China). After 1 week of acclimatization, mice were randomly divided into the Control, NC, Model, RRAH, RRAL and SILY (silymarin, positive control) groups. The Control group received distilled water, the NC group received a vehicle, and the RRAL and RRAH groups received 200 and 600 mg/kg (body weight) RRA, respectively.

RRA was administered orally to the mice for seven consecutive days. Then, except for the Control and NC groups, the other groups were induced as ALD models by administration of 45% (*v*/*v*) alcohol orally once a day by gavage with an amount of 10 mL/kg·(body weight) (equivalent to alcohol dose of 4.48 g/kg·d) for 14 consecutive days. Meanwhile, the SILY group received 500 mg/kg (body weight) silymarin (5304299-1g, Aladdin, Shanghai, China). Four hours after the final administration, all mice received 60% alcohol by gavage, except the Control and NC groups, which were given equal amounts of distilled water.

Loss and recovery of alcohol-induced righting reflex time and body weight were recorded. Upon completion of the experiment, all mice were euthanized, and their plasma, liver tissues, feces and colons were collected for subsequent experiments [30,31]. All experiments were conducted in compliance with the Institutional Guidelines and Animal Ordinance of the Kunming University of Science and Technology Animal Ethical Committee (PZWH (Dian) K2023-0025).

### 2.6. Assessment of the Drunken and Sober Time

Determination of drunk status: during drinking, mice were judged to be in a drunk status by looking down on their backs and up on their extremities to see if the positive fold reflex disappeared and persisted for 30 s. Awakening state determination: after entering an alcoholic state, mice were deemed to be awake if the observed reversion time was less than 30 s [32].

### 2.7. Liver Index and Staining

The mice livers were weighed, and the liver index was calculated as follows: Liver index = (livers mass/weight) × 100%. The fixed livers were dehydrated and embedded in paraffin to prepare 4 μm paraffin sections. Hematoxylin and Eosin (HE) staining was performed after dewaxing, hydration and so on. Meanwhile, the paraffin sections of the colon were made after dewaxing, hydration and other steps, incubation for 10 min in periodate solution and washing in distilled water, incubation for 45 min in Schiff reagent under dark conditions, rinsing with water, immersing in hydrochloric acid and ethanol for 30 s, rinsing with water for 5 min, drying and sealing the sheet. Images were captured with an optical biological microscope (Nikon, Minato-ku, Tokyo, Japan).

### 2.8. Liver Function and Cytokines Determination

The activity or content of aspartate aminotransferase (AST) (C010-2-1, Nanjing Jiancheng Bioengineering Institute, Nanjing, Jiangsu, China), superoxide dismutase (SOD) (A001-3-2, Nanjing Jiancheng Bioengineering Institute, Nanjing, Jiangsu, China), reactive oxygen species (ROS) (S0033S, Beyotime, Shanghai, China), S1P (ml062988, Mlbio, Shanghai, China), D-Lactate (D-LA) (ml974725, Mlbio, Shanghai, China), alanine aminotransferase (ALT) (C009-2-1, Nanjing Jiancheng Bioengineering Institute, Nanjing, Jiangsu, China), LPS (YJ037221, Shanghai Yuanji, Shanghai, China) and malondialdehyde (MDA) (S0131S, Beyotime, Shanghai, China) in the plasma or livers were measured with kits according to the manufacturer’s instructions.

### 2.9. TUNEL Assay

Liver tissues were rapidly harvested and embedded in the optimal cutting temperature compound (OCT) reagent (SAKURA, Chuo-Ku, Tokyo, Japan), and after that, they were sliced with freezing microtome (Leica, Am Leitz-Park, Wetzlar, Germany). Then, immunofluorescence staining was performed on 10 μm mouse liver cryosections. Apoptosis was detected with a TUNEL Assay Kit (C710, Beyotime, Shanghai, China) according to the manufacturer’s instructions. Images were captured with a fluorescence microscope (Nikon, Minato-ku, Tokyo, Japan) and analyzed by Image Pro Plus (6.0).

### 2.10. Gut Microbiota 16S rRNA Sequencing

Mice feces were collected for 16s rRNA sequencing analysis. Bacterial DNA was extracted, amplified and sequenced at Novogene Bioinformatics Technology Co., Ltd. (Tianjin, China). Genomic DNA was used to amplify the 16S rDNA V3-V4 region with barcoded primers, and construction of the PCR products library and sequencing was performed following the manufacturer’s instructions (Novogene Co., Ltd.).

### 2.11. Western Blot

The liver tissues were collected with the RIPA buffer (R0020, Solarbio, Beijing, China). The protein contents were detected by a Bicinchoninic acid (BCA) protein assay kit (P0010, Beyotime, Shanghai, China). Equal protein amounts were loaded for protein electrophoresis. The antibodies for proteins were purchased from Proteintech (Wuhan, Hubei, China): TLR4 (1:1000, 66350-1-Ig); IL-1β (1:1000, 168-1-AP); SPHK2 (1:3000, 17096-1-AP); BCL2 (1:1000, 66799-1-lg); NF-κB (1:2000, 10745-1-AP); and GAPDH (1:100,000, 60004-1-lg). The IgG-horseradish peroxidase (HRP) secondary antibodies (1:10,000) were incubated. Protein bands were exposed using a Novex™ ECL Chemiluminescent Kit (WP20005, Thermo Fisher Scientific, Waltham, MA, USA).

### 2.12. Statistical Analysis

Statistical analyses were conducted by GraphPad Prism v.8.0.1 (San Diego, CA, USA). The data were analyzed using a one-way analysis of variance (ANOVA), and the differences between the groups were determined using a Tukey’s post hoc test. A statistically significant difference was considered for *p* < 0.05, and the data were represented as the mean ± SEM.

## 3. Results

### 3.1. Extraction and Identification of Red Rice SEED Coat

**Figure 1:**
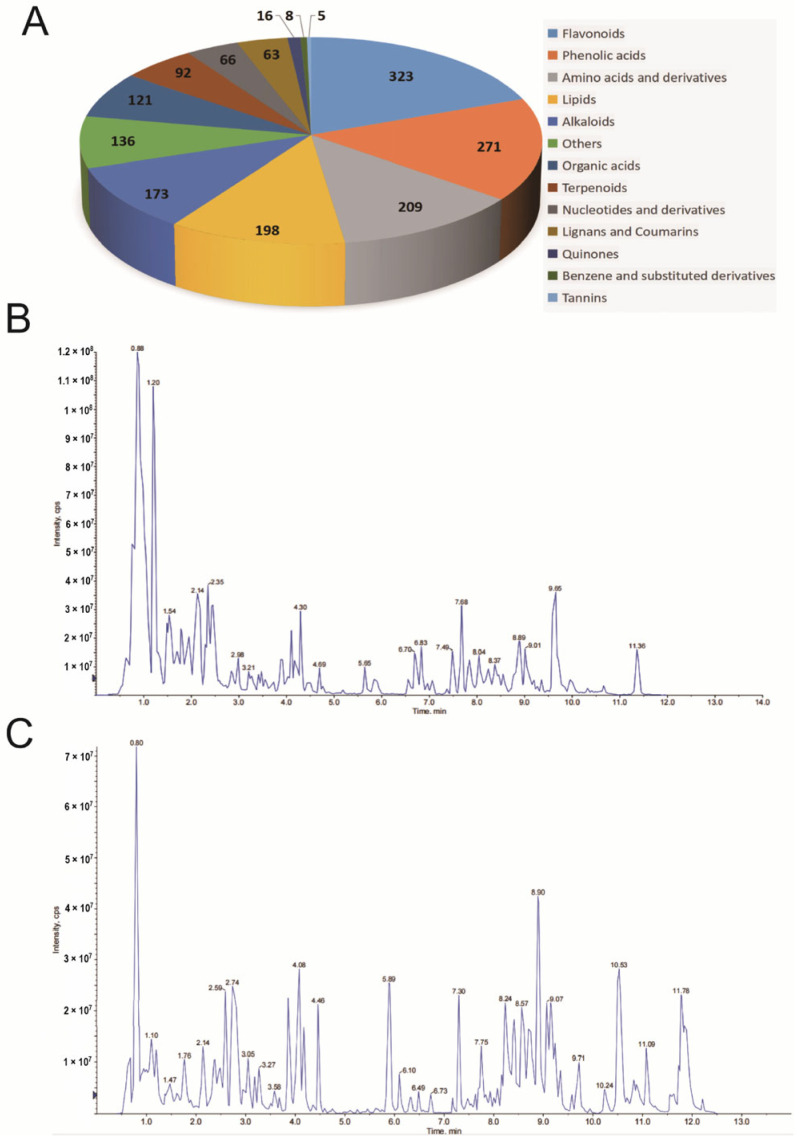
Analysis of RRA chemical compositions. (**A**) 1681 components were identified in RRA by widely targeted components analysis. (**B**) The total ion current diagram of RRA samples in positive ionization mode. (**C**) The total ion current diagram of RRA samples in negative ionization mode.

To identify the chemical compositions of RRA, a widely targeted components analysis was performed. A total of 1681 components were identified, including 323 flavonoids, 271 phenolic acids, 209 amino acids and derivatives, 198 lipids, 173 alkaloids, 121 organic acids, 92 terpenoids, 66 nucleotides and derivatives, 63 lignans and coumarins, 16 quinones, 8 benzene and its derivatives, 5 tannins and 136 other metabolites (i.e., vitamins, Saccharides, etc.) (Figure 1A). The total ion current diagram of RRA samples in positive (Figure 1B) and negative (Figure 1C) ionization modes are shown.

### 3.2. Effects of RRA on Body Weight, Postural Reflex and Liver Index of ALD Mice

**Figure 2:**
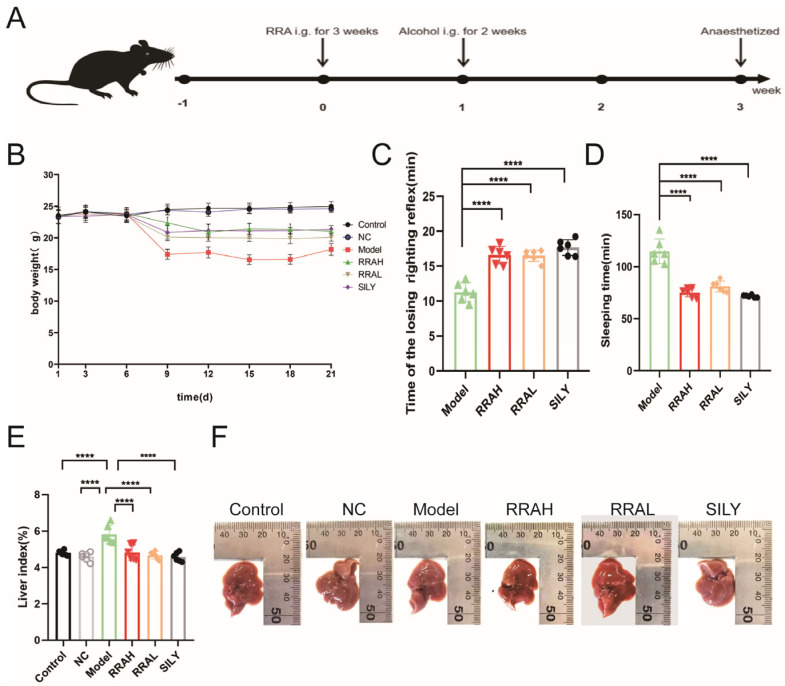
Effects of RRA on body weight, postural reflex and liver index of ALD mice. (**A**) Schematic illustration of animal experiment. (**B**) Body weight of mice. (**C**) The function of RRA on loss of righting reflex time of mice. (**D**) The effects of RRA on sleeping time of mice. (**E**) The effects of RRA on liver index of mice. (**F**) The effects of RRA on liver morphology. RRAH, 600 mg/kg·body weight; RRAL, 200 mg/kg·body weight. Data are shown as the mean ± SEM, *n* = 6. **** *p* < 0.0001.

According to the scheme of animal experiments (Figure 2A), the weight loss in RRAH, RRAL and SILY mice gradually slowed, while weight gain was slow in Control and NC mice but was significantly decreased in the Model mice (Figure 2B). The time taken for tolerance (time of loss of righting reflex, LORR) of mice treated with RRAH, RRAL and SILY was more prolonged compared with the Model group (Figure 2C). These results showed that compared with Model group, the sleep times of RRAH, RRAL and SILY groups were obviously cut down (Figure 2D). The liver index was higher in the Model group compared to the Control and NC groups, but lower in the RRAL, RRAH and SILY groups compared to the Model group (Figure 2E). The livers of the Control and NC groups were soft and bright reddish-brown, the ALD Model livers were dark red and had obvious lesions, but the livers of the RRAL, RRAH and SILY groups were significantly improved (Figure 2F).

### 3.3. RRA Alleviated Liver Pathological Injury and Oxidative Stress, and Improved Liver Function in ALD Mice

**Figure 3:**
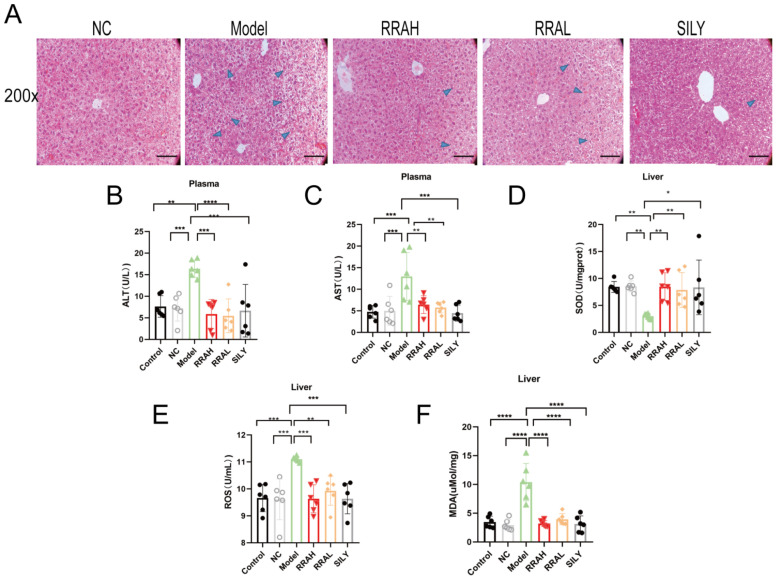
RRA alleviated liver pathological injury and oxidative stress, and improved liver function in ALD mice. (**A**) Liver HE staining: the arrows in blue mark pathologic lesions. Scale plates were 100 μm. (**B**–**F**) The effects of RRA on ALT, AST, SOD, ROS and MDA. RRAH, 600 mg/kg·body weight; RRAL, 200 mg/kg·body weight. Data are shown as the mean ± SEM, *n* = 6. * *p* < 0.05, ** *p* < 0.01, *** *p* < 0.001, and **** *p* < 0.0001.

HE staining of the livers showed that RRA treatment alleviated the livers pathological injury in ALD mice, which were similar to the SILY group (Figure 3A). The ALT and AST levels of Model mice livers were elevated, and RRAL, RRAH and SILY could significantly reduce them (Figure 3B,C). SOD levels of Model group livers were lower than Control and NC groups, but administration of RRAL, RRAH and SILY significantly increased the SOD level (Figure 3D). In the ALD mouse model, ROS and MDA levels were notably increased; comparatively, RRAH, RRAL and SILY treatment significantly decreased ROS and MDA levels (Figure 3E,F).

### 3.4. RRA May Ameliorate ALD by Regulating SPHK2 as Predicted by Bioinformatics

**Figure 4:**
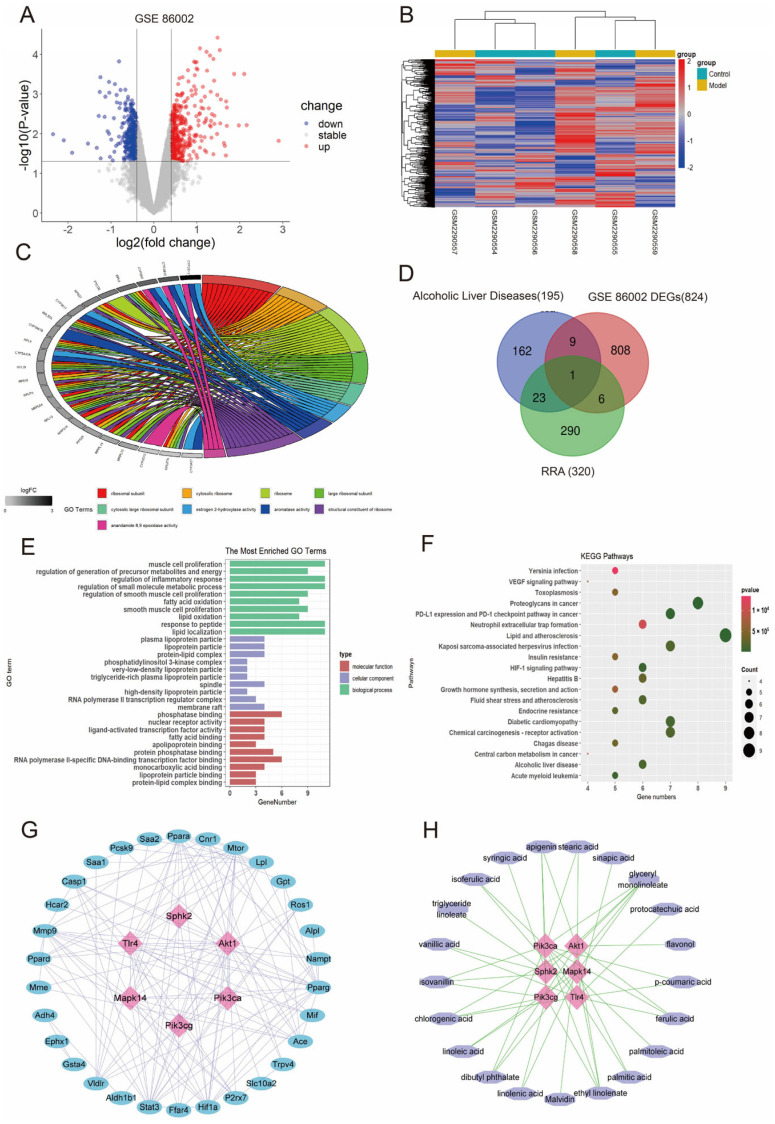
RRA may ameliorate ALD by regulating SPHK2 as determined by bioinformatic analysis. (**A**) Volcano plot of DEGs between ALD and Control groups in GSE86002. The red points demonstrate overexpression genes, blue points demonstrate down-expression genes and gray points demonstrate stable genes (*p* < 0.05). (**B**) Heatmap of DEGs between ALD and Control groups in GSE86002. (**C**) GO analysis of DEGs (Top 9 enriched terms). (**D**) Venn diagram of DEGs of GSE86002, potential target genes of RRA, and ALD-related genes. (**E**) GO analysis of overlapping genes. (**F**) Top 20 relevant pathways: analysis of KEGG pathways in overlapping genes. (**G**) PPI net of overlapping genes. The blue and pink points demonstrate overlapping genes, the edges demonstrate the interactivities of these genes. (**H**) Pharmacological targets and RRA compounds net against ALD. Pink points demonstrate the protein targets, purple points demonstrate the compounds, and edges demonstrate the interactivities of them.

In GSE86002, 824 DEGs of ALD were obtained, which contained 419 upregulated and 405 downregulated mRNAs (Figure 4A). The DEGs were easy to distinguish between Control and ALD groups in GSE86002 (Figure 4B). The top nine terms of DEGs GO annotation are shown in Figure 4C. We picked out 26 compounds in the result of widely targeted components analysis (Table S1). A total of 320 potential targets of action onto 26 compounds were obtained in the Swiss Target database (Table S2). A total of 195 ALD-associated genes were retrieved in the DisGeNET (Table S3). A total of 39 overlapping genes were filtrated among RRA target genes, ALD-associated genes and GSE86002 DEGs (Figure 4D). Next, exploration and enrichment of overlapping genes from three domains, including molecular function, cellular components, and biological processes, was performed by GO analysis annotation and revealed the top 10 of them (Figure 4E). The top 20 KEGG pathways are revealed in Figure 4F, including alcoholic liver disease, diabetic cardiomyopathy and so on. Overlapping genes PPI was ascertained by STRING, and Cytoscape was used to construct the network, which consisted of 36 nodes and 136 edges (Figure 4G). In addition, RRA compounds and the targets network of red rice seed coat against ALD was built with Cytoscape (Figure 4H). Taken together with the results of the above analysis and the literature reports, SPHK2 was chosen as a prospective target.

### 3.5. RRA Restored Intestinal Barrier in ALD Mice by Upregulating SPHK2/S1P

**Figure 5:**
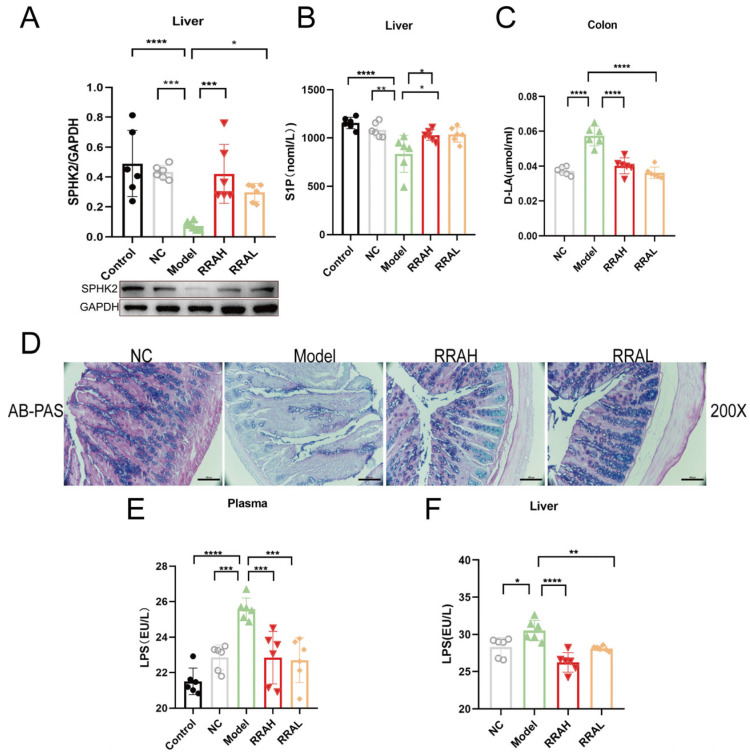
RRA restored the intestinal barrier in ALD mice by upregulating SPHK2/S1P. (**A**–**C**) The effects of RRA on SPHK2, SIP and D-LA levels in mice livers. (**D**) Representative AB-PBS staining of mice colons. Scale bars were 200 μm. (**E**,**F**) The effects of RRA on the plasma LPS and liver LPS contents. RRAH, 600 mg/kg·body weight; RRAL, 200 mg/kg·body weight. Data are shown as the mean ± SEM, *n* = 6. * *p* < 0.05, ** *p* < 0.01, *** *p* < 0.001, and **** *p* < 0.0001.

To confirm the effects of RRA on the intestinal barrier via SPHK2/S1P in ALD mice, the levels of associated factors were detected in liver tissues. In ALD Model groups, SPHK2 expression was downregulated, but administration of RRAL and RRAH upregulated SPHK2 expression (Figure 5A). Consequently, S1P levels were reduced in Model groups, comparatively, and RRAH and RRAL treatment obviously enhanced them (Figure 5B). Compared with the Model group, D-LA levels were significantly reduced in the colons of mice treated with RRAL and RRAH (Figure 5C). Colon AB−-PAS stains are shown in Figure 5D. It could be observed from the AB-PAS stains that mucin secretion increased in RRA groups, especially in the RRAH group. Further analysis showed that mucin density and the degree of mucin alignment significantly reduced in the ALD model group. The levels of LPS in ALD model plasma were notably increased by damaging the intestinal barrier, and RRAL and RRAH treatment remarkably reduced LPS in the plasma (Figure 5E). Thus, LPS showed a marked increase in the ALD model liver via the gut–liver axis, and administration of RRAH and RRAL significantly decreased LPS in the liver (Figure 5F). Taken together, RRA maintained the intestinal barrier and decreased the LPS level in the ALD mice by upregulating SPHK2/S1P.

### 3.6. RRA Attenuated Inflammation and Inhibited Apoptosis in ALD Mice Livers

**Figure 6:**
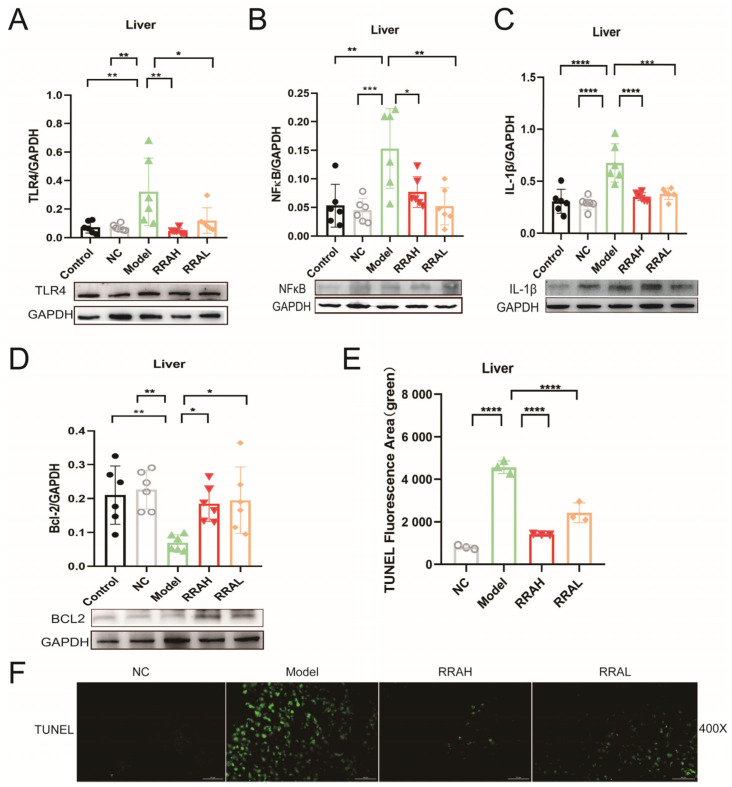
RRA attenuated inflammation and inhibited apoptosis in ALD mice liver. (**A**–**D**) The expressions of TLR4, NF-κB, IL-1β and BCL2. (**E**,**F**) Immunofluorescent of apoptosis in liver tissues. Green immunofluorescent was representative for apoptotic cells in liver tissues. Scale bars were 50 μm. RRAH, 600 mg/kg·body weight; RRAL, 200 mg/kg·body weight. Data are shown as the mean ± SEM, *n* = 6. * *p* < 0.05, ** *p* < 0.01, *** *p* < 0.001, and **** *p* < 0.0001.

Through the gut–liver axis, LPS could activate the hepatocyte TLR4 signaling pathway, which caused inflammatory responses and apoptosis in the ALD model liver. TLR4, NF-κB and IL-1β were remarkably increased in the Model groups; however, the RRAH and RRAL treatment all decreased them (Figure 6A–C). Differently, BCL-2 level was significantly decreased in the Model group, administration of RRAH and RRAL could all sharply increase the BCL-2 expression (Figure 6D). To verify RRA effects on the hepatocyte apoptosis, TUNEL staining was operated in the ALD mouse model. In Model groups, apoptosis cells with green fluorescent were significantly increased; comparatively, RRAH and RRAL treatment decreased apoptosis cells (Figure 6E,F). These results clearly stated that RRA attenuated inflammation and inhibited apoptosis in ALD mice liver by the gut–liver axis.

### 3.7. RRA Improved Gut Microbiota Composition in ALD Mice

**Figure 7:**
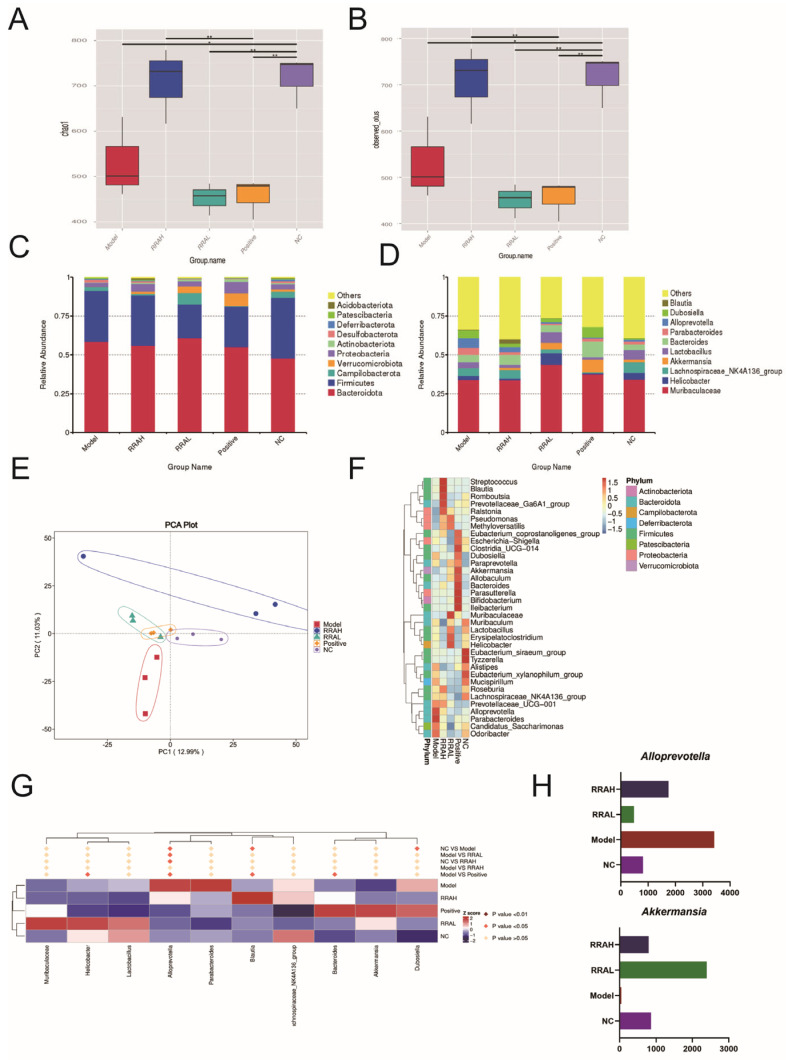
RRA improved gut microbiota composition in ALD mice. (**A**) The Chao 1 index. (**B**) The Observed OTUs index. (**C**) Gut microbiota compositions at phylum levels (Top 10). (**D**) Gut microbiota compositions at genus levels (Top 10). (**E**) PCA analysis results. (**F**) Heatmap of top 35 gut bacteria at genus levels. (**G**) Clustering heatmap of species abundance at genus levels. (**H**) Relative abundances of *Akkermansia* and *Alloprevotella*, *n* = 3. RRAH, 600 mg/kg·body weight; RRAL, 200 mg/kg·body weight. Data are shown as the mean ± SEM * *p* < 0.05, ** *p* < 0.01.

To further explore the mechanisms of RRA on ALD model mice, we analyzed the microbiota 16s rDNA of mice feces. Results showed that the Chao 1 and Observed OTUs indexes were remarkably decreased in the Model group, and RRAH treatment restored those tendencies (Figure 7A,B). Results of top 10 intestinal bacteria at phyla and genera are shown in Figure 7C,D, respectively. Moreover, the similarities of gut microbiota composition were analyzed by PCA. Results showed that microflora in the ALD Model was obvious to distinguish from that in NC group, and RRAH and RRAL treatment improved the microflora of ALD mice (Figure 7E). Results of the top 35 intestinal bacteria at genera are shown in Figure 7F. To identify the important bacteria in ALD mice after RRA administration, the MetaStat method was performed in genus levels (Figure 7G). *Akkermansia* and *Alloprevotella* were found as key bacterium by MetaStat analysis (Figure 7H). Specifically, *Akkermansia* was associated with the intestinal barrier [33], and *Alloprevotella* was closely related to relieved intestinal inflammation and reduced intestinal diseases [34,35].

## 4. Discussion

Alcohol misuse is the third largest risk factor for human health, and ALD is a primary factor of liver disease worldwide. Therefore, it is meaningful to explore the active ingredients in red rice coat that can alleviate clinical ALD. Then, ingredients of RRA were extracted and identified, which were rich in active substances, such as flavonoids, phenolic acids, alkaloids, terpenoids and so on. According to previous reports, natural polyphenol and flavonoids from plants have antioxidant capacity and anti-inflammatory properties [36]. In the same way, RRA treatment notably improved ALD by alleviating oxidative stress, reducing inflammation, inhibiting apoptosis, improving gut microbiota and restoring the intestinal barrier in vivo. Our work revealed that red rice seed coat holds potential as a functional food or therapeutic agent for the prevention and treatment of human ALD.

A total of 824 DEGs of ALD were identified in GSE86002, which involved 419 upregulated and 405 downregulated mRNAs. A total of 320 predictive molecular targets might respond to RRA treatment, 195 ALD-associated genes were obtained, and 39 overlapping genes were screened from DEGs, ALD-associated genes and predictive molecular targets of RRA. The molecular function, cellular signaling pathways and biological processes were performed by GO and KEGG analysis. The network of overlapping genes PPI and targets vs. compounds network were constructed to further illustrate interaction modes. Taken together, we picked out SPHK2 as a potential key target for RRA against ALD. Afterward, we verified the inference through experiments in the ALD mice model.

We found that RRA could significantly reduce the adverse effects of alcohol on ALD mice body weight, postural reflex and liver index, which was consistent with the improvement effect of ALD [37]. By analyzing the weight change in mice, the effect of RRA on the weights of mice with ALD could be estimated. The duration of the righting reflex and liver index reflected that the drunken state and the damage to the liver were restored by RRA, and that RRA could restore liver function and systemic functional state. Oxidative stress is a key process through ALD pathogenesis [38]; at the same time, we found that RRA treatment could reduce the AST, ALT, ROS and MDA levels and raise SOD in ALD mice. These observations revealed that RRA could improve the ability to alleviate oxidative stress and enhance liver function in ALD mice.

The intestine and liver are key organs for nutrient absorption and metabolism, and there is a wide and close interaction between the intestine and liver, which was known as the gut–liver axis. Given that 70% of the blood flows from the intestine to the liver, impairment of intestinal integrity leads to transient endotoxaemia, thereby promoting liver inflammation, termed intestinal leak syndrome [39]. Increased intestinal permeability due to intestinal barrier injury is the main cause of inflammatory response and apoptosis in the ALD liver [40]. TLR4 mainly mediated the inflammatory response and was involved in biological processes such as immunity, but it also plays a major role in the pathogenesis of ALD [41]. The SPHK2 protein and its positively regulated SIP levels were both reduced in the Model mice and were increased by RRA treatment. S1P is a biologically active hemolysophospholipid, which has important biological functions, such as cell adhesion, barrier regulation, proliferation, differentiation, migration and survival. D-LA is a key indicator of intestinal permeability, and elevated levels indicate damage to the intestinal barrier [39,42]. Additionally, the mucin of the intestinal barrier in AB-PAS staining in model mice showed an obvious density reduction and arrangement disorder [43].

In ALD mice, the intestinal barrier is disrupted and the LPS level in the plasma is increased. LPS triggers TLR4 of liver cells and activates the downstream NFκB signaling pathways, and activation of NFκB can elevate expression of inflammatory cytokines such as IL-1β [44]. In this study, alcohol intake induced intestinal barrier damage and increased plasma LPS, which led to downstream harmful effects by the TLR4 signaling pathway. RRA effectively cut down on the serum LPS level whilst inhibiting the TLR4/NFκB pathway. The NFκB phosphorylation state and immunofluorescence staining of p-NFκB subcellular localization will be examined in future works. Furthermore, the TUNEL confirmed that RRA could attenuate apoptosis by promoting BCL2 in ALD.

**Figure 8:**
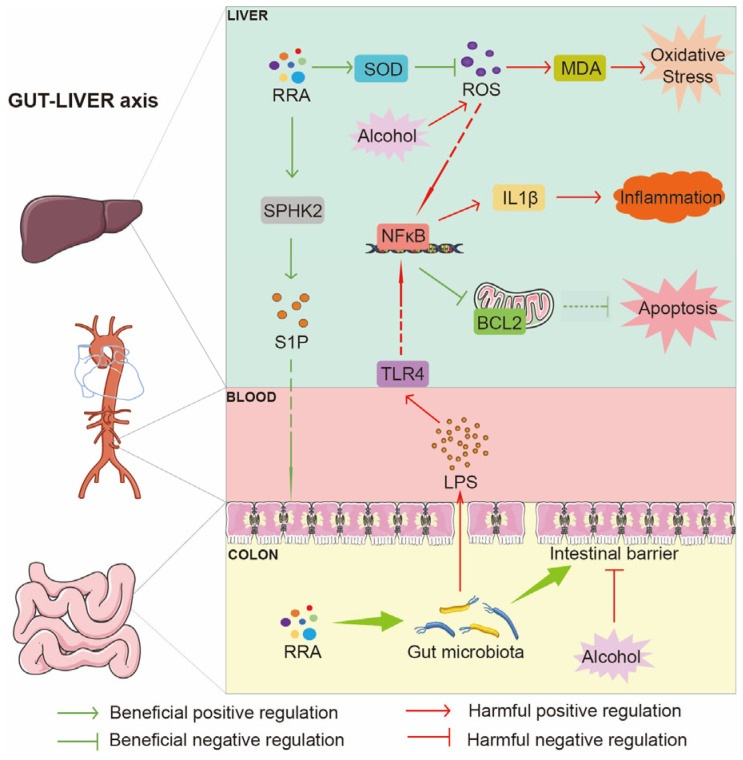
The mechanism of RRA-ameliorated alcoholic liver disease.

Gut microbiota and its metabolites produced a marked effect on monitoring the host-specific physiological process and sustaining homeostasis [45]. Newly, evolving confirmation holds that gut microbiota maintains the intestinal barrier. Some studies exhibited that the species constitution and metabolic profiles of gastroenteric microbiota in ALD mice are unbalanced, and the LPS from pathogenic bacteria could cause a disturbance to epithelial mechanical barrier integrity [46]. Administration of RRA improved the balance of bacteria in an ALD colon, especially *Akkermansia* and *Alloprevotella*, which have the function of maintaining the intestinal barrier [35,47,48]. These results then further suggested that RRA targeting of SPHK2 can ameliorate ALD in mice by repairing intestinal barrier damage and reducing the plasma LPS level via the LPS/TLR4/NFκB pathway and intestinal microbiota (Figure 8).

Red rice seed coat may regulate multiple targets and signaling pathways because of its plentiful active substances, such as flavonoids, phenolic acids and so on. In the future, different fractions of RRA will be isolated and purified, effects of various fractions on ALD will be investigated to further explore the mechanisms, and we will evaluate the most active ingredients and related signaling pathways.

## 5. Conclusions

In ALD model mice, RRA treatment increased SPHK2 and S1P levels, improved gut microbiota composition, and restored the intestinal barrier; meanwhile, it decreased LPS levels of the plasma and liver and cut down the TLR4/NF-κB pathway, whilst alleviating liver pathological injury and oxidative stress, and attenuating inflammation and apoptosis to enhance liver function. To sum up, RRA targeting SPHK2 can ameliorate ALD of mice by repairing intestinal barrier damage and reducing liver LPS levels via the TLR4/NF-κB pathway and intestinal microbiota. Red rice seed coat might be considered to become a reassuring resource for functional food.

## Data Availability

The data supporting this study’s findings are available upon request.

## Supplementary Materials

The following supporting information can be downloaded at: https://www.mdpi.com/article/10.3390/nu15194176/s1, Table S1: Compounds of RRA; Table S2: Potential molecular targets of compounds in RRA; Table S3: Summary of ALD-Gene Associations.

## Abbreviations

AB-PAS	Periodic acid Schiff (PAS) and Alcian blue (AB) stain
ALD	Alcoholic liver disease
ALT	Alanine aminotransferase
AST	Aspartate aminotransferase
BCA	Bicinchoninic acid
BCL2	B-cell lymphoma-2
DEGs	Differentially expressed genes
D-LA	D-Lactate
ECL	Enhanced chemiluminescence
GEO	Gene Expression Omnibus
GO	Gene Ontology
HE	Hematoxylin and eosin
HRP	Horseradish peroxidase
IgG	Immunoglobulin G
IL-1β	Interleukin-1β
KEGG	Kyoto Encyclopedia of Genes and Genomes
LORR	Loss of righting reflex
LPS	Lipopolysaccharides
MDA	Malondialdehyde
MRM	Multiple reaction monitoring mode
NF-κB	Nuclear factor kappa B
OCT	Optimal cutting temperature compound
PPI	Protein–protein interaction
QQQ	Triple quadrupole mass
ROS	Reactive oxygen species
RRA	Red rice seed coat extract
S1P	Phingosine-1-phosphate
SOD	Superoxide dismutase
SPHK2	Sphingosine kinase 2
TLR4	Toll-like receptor 4
TUNEL	TdT-mediated dUTP nick end labeling
