# Supplementary material for: Red Rice Seed Coat Targeting SPHK2 Ameliorated Alcoholic Liver Disease via Restored Intestinal Barrier and Improved Gut Microbiota in Mice
Source: Nutrients. 2023 Sep 27;15(19):4176. doi: 10.3390/nu15194176 (PMC10574211; doi:10.3390/nu15194176)
Supplement: Supplementary file 1 [file nutrients-15-04176-s001.zip › nutrients-2618389-supplementary.pdf]

Table S1. Compounds of RRA. 26 compounds of RRA were selected in the result of widely targeted component analysis.

**Compounds**	**Ionization model**	**Class I**	**Class II**	**Q1 (Da)**	**Q3 (Da)**	**CAS**
7-Hydroxyflavone	[M+H]+	Flavonoids	Flavones	137.02	238.063	6665-86-7
Ferulic acid	[M-H]-	Phenolic acids	Phenolic acids	134.04	194.0579	537-98-4
Sinapic acid	[M-H]-	Phenolic acids	Phenolic acids	193.02	224.0685	530-59-6
3,4-Dihydroxybenzoic acid (Protocatechuic acid)	[M-H]-	Phenolic acids	Phenolic acids	109.03	154.0266	99-50-3
Syringic acid	[M-H]-	Phenolic acids	Phenolic acids	123.01	198.0528	530-57-4
Chlorogenic acid (3-O-Caffeoylquinic acid)(3-O-Caffeoylquinic acid)	[M-H]-	Phenolic acids	Phenolic acids	191.06	354.0951	327-97-9
Linoleic acid	[M-H]-	Lipids	Free fatty acids	59.01	280.2402	60-33-3
α-Linolenic Acid	[M-H]-	Lipids	Free fatty acids	277.22	278.2246	463-40-1
Apigenin; 4',5,7-Trihydroxyflavone	[M+H]+	Flavonoids	Flavones	153.02	270.0528	520-36-5
Palmitic acid	[M-H]-	Lipids	Free fatty acids	237.22	256.2402	57-10-3
Docosanoic acid (Behenic acid)	[M-H]-	Lipids	Free fatty acids	183.17	340.3341	112-85-6
Epicatechin	[M+H]+	Flavonoids	Flavanols	139.04	290.079	490-46-0
IsoFerulic Acid	[M-H]-	Phenolic acids	Phenolic acids	134.0374	194.0579	25522-33-2
Vanillic acid	[M-H]-	Phenolic acids	Phenolic acids	108.02	168.0423	121-34-6
Isovanillin	[M+H]+	Phenolic acids	Phenolic acids	65.04	152.0473	621-59-0
1-Linoleoylglycerol	[M+H]+	Lipids	Glycerol ester	263.24	354.277	2277-28-3
Methyl linolenate	[M+H]+	Lipids	Free fatty acids	95.09	292.2402	301-00-8
Diisobutyl phthalate	[M+H]+	Phenolic acids	Phenolic acids	149.0233	278.1518	84-69-5
Hydroperoxylinoleic acid	[M-H]-	Lipids	Free fatty acids	223.17	312.2301	34444-18-3
Elaidic Acid	[M-H]-	Lipids	Free fatty acids	281.25	282.2559	112-79-8
(7Z)-Hexadecenoic acid	[M-H]-	Lipids	Free fatty acids	235.21	254.2246	2416-19-5
10-Hydroxystearic Acid	[M-H]-	Lipids	Free fatty acids	141.13	300.2664	638-26-6
Hydroxyicosanoic Acid	[M-H]-	Lipids	Free fatty acids	59.01	328.2977	-
Catechin-catechin-catechin	[M-H]-	Flavonoids	Flavanols	407.1	866.2058	-
p-Coumaric acid ethyl ester	[M+H]+	Phenolic acids	Phenolic acids	147.04	192.0786	7362-39-2
Malvidin-3-O-galactoside (Primulin)	[M]+	Flavonoids	Anthocyanidins	331.08	493.1341	30113-37-2

Table S2. Potential molecular targets of compounds in RRA. In Swiss Target Prediction database, 320 potential molecular targets of 26 compounds were collected.

Compound	Target Gene	ChEMBL ID	Uniprot ID	Probability*	Known actives (3D/2D)
7-Hydroxyflavone	Adora1	CHEMBL3688	Q60612	0.198808518	258 / 23
7-Hydroxyflavone	Adora2a	CHEMBL2115	Q60613	0.198808518	194 / 11
7-Hydroxyflavone	Hsd17b2	CHEMBL1914270	P51658	0.117056358	12 / 2
7-Hydroxyflavone	Alox5	CHEMBL5211	P48999	0.108018051	45 / 47
7-Hydroxyflavone	Tnks	CHEMBL3232702	Q6PFX9	0.108018051	14 / 28
7-Hydroxyflavone	Bche	CHEMBL2528	Q03311	0.098947479	12 / 3
7-Hydroxyflavone	Ca7	CHEMBL2216	Q9ERQ8	0.080792387	48 / 8
7-Hydroxyflavone	Ache	CHEMBL3198	P21836	0.080792387	37 / 24
7-Hydroxyflavone	Tnks2	CHEMBL3232703	Q3UES3	0.080792387	16 / 12
7-Hydroxyflavone	Tyr	CHEMBL5346	P11344	0.080792387	0 / 3
7-Hydroxyflavone	Alox12	CHEMBL3225	P39655	0.071715932	11 / 9
7-Hydroxyflavone	Abcb1a	CHEMBL2573	P21447	0.071715932	11 / 42
7-Hydroxyflavone	Abcb1b	CHEMBL3467	P06795	0.071715932	11 / 39
7-Hydroxyflavone	Mmp9	CHEMBL2214	P41245	0.062621967	11 / 2
7-Hydroxyflavone	Mmp2	CHEMBL3095	P33434	0.062621967	16 / 2
7-Hydroxyflavone	Gsk3b	CHEMBL1075321	Q9WV60	0.062621967	139 / 8
7-Hydroxyflavone	Maoa	CHEMBL3681	Q64133	0.062621967	74 / 25
7-Hydroxyflavone	Aurkb	CHEMBL1075275	O70126	0.062621967	25 / 4
7-Hydroxyflavone	Cdk1	CHEMBL4084	P11440	0.062621967	34 / 10
7-Hydroxyflavone	Ar	CHEMBL3056	P19091	0.062621967	25 / 16
7-Hydroxyflavone	Maob	CHEMBL3050	Q8BW75	0.062621967	141 / 29
7-Hydroxyflavone	Pla2g1b	CHEMBL4378	Q9Z0Y2	0.062621967	0 / 1
7-Hydroxyflavone	Mmp13	CHEMBL3638350	P33435	0.062621967	13 / 1
7-Hydroxyflavone	Egfr	CHEMBL3608	Q01279	0.062621967	460 / 24
7-Hydroxyflavone	Prkdc	CHEMBL2176779	P97313	0.053556076	36 / 8
7-Hydroxyflavone	Bace1	CHEMBL4593	P56818	0.053556076	269 / 12
7-Hydroxyflavone	Lck	CHEMBL2480	P06240	0.053556076	49 / 3
7-Hydroxyflavone	Esr2	CHEMBL2995	O08537	0.053556076	19 / 33
7-Hydroxyflavone	Pik3cg	CHEMBL2189158	Q9JHG7	0.053556076	57 / 1
7-Hydroxyflavone	Abcc1	CHEMBL2532	O35379	0.053556076	1 / 11
7-Hydroxyflavone	F2	CHEMBL1075308	P19221	0.053556076	32 / 3
7-Hydroxyflavone	Ca13	CHEMBL2186	Q9D6N1	0.053556076	27 / 1
7-Hydroxyflavone	Akr1c21	CHEMBL1075270	Q91WR5	0.053556076	2 / 1
7-Hydroxyflavone	Alk	CHEMBL5771	P97793	0.053556076	42 / 3
7-Hydroxyflavone	Fyn	CHEMBL4517	P39688	0.053556076	8 / 1
7-Hydroxyflavone	Akt1	CHEMBL5859	P31750	0.053556076	12 / 4
7-Hydroxyflavone	Csnk2a1	CHEMBL3537	Q60737	0.053556076	15 / 2
7-Hydroxyflavone	Insr	CHEMBL3187	P15208	0.053556076	8 / 1
7-Hydroxyflavone	Mapk3	CHEMBL5510	Q63844	0.053556076	1 / 1
7-Hydroxyflavone	Pygl	CHEMBL3008	Q9ET01	0.053556076	11 / 1

7-Hydroxyflavone	Ptgs2	CHEMBL4321	Q05769	0.053556076	95 / 44
7-Hydroxyflavone	Esr1	CHEMBL3065	P19785	0.053556076	14 / 34
7-Hydroxyflavone	Pparg	CHEMBL2459	P37238	0.053556076	0 / 3
7-Hydroxyflavone	Top1	CHEMBL2814	Q04750	0.053556076	0 / 1
7-Hydroxyflavone	Tacr2	CHEMBL2813	P30549	0.053556076	2 / 1
7-Hydroxyflavone	Parp1	CHEMBL3740	P11103	0.053556076	9 / 0
7-Hydroxyflavone	Ctsl	CHEMBL5291	P06797	0.053556076	5 / 0
7-Hydroxyflavone	Drd2	CHEMBL3427	P61168	0.053556076	18 / 59
7-Hydroxyflavone	Mcl1	CHEMBL5768	P97287	0.053556076	2 / 1
7-Hydroxyflavone	Cd38	CHEMBL3425388	P56528	0.053556076	23 / 2
7-Hydroxyflavone	Tyms	CHEMBL3160	P07607	0.053556076	80 / 0
7-Hydroxyflavone	Plg	CHEMBL1075299	P20918	0.053556076	0 / 3
7-Hydroxyflavone	Grm5	CHEMBL1641352	Q3UVX5	0.053556076	263 / 0
7-Hydroxyflavone	Parp2	CHEMBL2794	O88554	0.053556076	6 / 1
7-Hydroxyflavone	Jak3	CHEMBL5250	Q62137	0.053556076	219 / 0
7-Hydroxyflavone	Gsk3a	CHEMBL2176843	Q2NL51	0.053556076	27 / 0
7-Hydroxyflavone	Prkcd	CHEMBL2560	P28867	0.053556076	3 / 0
7-Hydroxyflavone	Prkch	CHEMBL4992	P23298	0.053556076	1 / 0
7-Hydroxyflavone	Alpl	CHEMBL2660	P09242	0.053556076	40 / 0
7-Hydroxyflavone	Aurka	CHEMBL2211	P97477	0.053556076	36 / 0
7-Hydroxyflavone	Impdh2	CHEMBL3169	P24547	0.053556076	3 / 0
7-Hydroxyflavone	Htr1a	CHEMBL3737	Q64264	0.053556076	20 / 0
7-Hydroxyflavone	Slc6a3	CHEMBL2799	Q61327	0.053556076	9 / 0
7-Hydroxyflavone	Nos2	CHEMBL3464	P29477	0.053556076	79 / 3
7-Hydroxyflavone	Tlr7	CHEMBL6085	P58681	0.053556076	7 / 0
7-Hydroxyflavone	Mdm2	CHEMBL3600279	P23804	0.053556076	9 / 0
7-Hydroxyflavone	Mapk9	CHEMBL2034797	Q9WTU6	0	73 / 0
7-Hydroxyflavone	Ret	CHEMBL2034799	P35546	0	46 / 0
7-Hydroxyflavone	Cnr1	CHEMBL3037	P47746	0	117 / 0
7-Hydroxyflavone	Cnr2	CHEMBL5373	P47936	0	77 / 0
7-Hydroxyflavone	Dyrk1a	CHEMBL4750	Q61214	0	67 / 0
7-Hydroxyflavone	Dhodh	CHEMBL2991	O35435	0	7 / 0
7-Hydroxyflavone	Sigmar1	CHEMBL3465	O55242	0	2 / 64
7-Hydroxyflavone	Hsd11b1	CHEMBL3910	P50172	0	48 / 0
7-Hydroxyflavone	Pik3ca	CHEMBL2499	P42337	0	122 / 0
7-Hydroxyflavone	Mtor	CHEMBL1255165	Q9JLN9	0	67 / 0
7-Hydroxyflavone	Ctsk	CHEMBL1075277	P55097	0	42 / 0
7-Hydroxyflavone	Pde10a	CHEMBL1795126	Q8CA95	0	208 / 0
7-Hydroxyflavone	Ptpn1	CHEMBL3336	P35821	0	7 / 14
7-Hydroxyflavone	Kcna3	CHEMBL4818	P16390	0	1 / 3
7-Hydroxyflavone	Tgfbr1	CHEMBL2021750	Q64729	0	40 / 0
7-Hydroxyflavone	Adora2b	CHEMBL2237	Q60614	0	63 / 0
7-Hydroxyflavone	Pdgfrb	CHEMBL2749	P05622	0	5 / 0
7-Hydroxyflavone	Kdr	CHEMBL3337	P35918	0	6 / 0

7-Hydroxyflavone	Flt1	CHEMBL3516	P35969	0	53 / 0
7-Hydroxyflavone	Braf	CHEMBL2331061	P28028	0	54 / 0
7-Hydroxyflavone	Tspo	CHEMBL2149	P50637	0	98 / 0
7-Hydroxyflavone	Abhd6	CHEMBL5010	Q8R2Y0	0	2 / 0
7-Hydroxyflavone	Mc4r	CHEMBL3719	P56450	0	3 / 0
7-Hydroxyflavone	Ctsb	CHEMBL5187	P10605	0	12 / 0
7-Hydroxyflavone	Fasn	CHEMBL1795189	P19096	0	3 / 0
7-Hydroxyflavone	Chrm1	CHEMBL3733	P12657	0	20 / 0
7-Hydroxyflavone	Stat3	CHEMBL5402	P42227	0	18 / 0
7-Hydroxyflavone	Taar1	CHEMBL4908	Q923Y8	0	38 / 0
7-Hydroxyflavone	Raf1	CHEMBL3804748	Q99N57	0	9 / 0
7-Hydroxyflavone	Ccr1	CHEMBL3872	P51675	0	6 / 0
7-Hydroxyflavone	Ccr2	CHEMBL5412	P51683	0	6 / 0
7-Hydroxyflavone	Ido1	CHEMBL1075294	P28776	0	22 / 0
7-Hydroxyflavone	Ephx2	CHEMBL4140	P34914	0	11 / 0
7-Hydroxyflavone	Bace2	CHEMBL3638357	Q9JL18	0	68 / 0
Malvidin-3-O-galactoside (Primulin)*	Oprd1	CHEMBL3222	P32300	0.104671941	0 / 5
Malvidin-3-O-galactoside (Primulin)*	Ptgs1	CHEMBL2649	P22437	0.104671941	0 / 3
Malvidin-3-O-galactoside (Primulin)*	Pde4b	CHEMBL2272	Q8VBU5	0.104671941	0 / 1
Malvidin-3-O-galactoside (Primulin)*	Oprm1	CHEMBL2858	P42866	0.104671941	0 / 1
Malvidin-3-O-galactoside (Primulin)*	Kit	CHEMBL2034798	P05532	0.104671941	0 / 2
Malvidin-3-O-galactoside (Primulin)*	Hdac6	CHEMBL2878	Q9Z2V5	0	0 / 1
Malvidin-3-O-galactoside (Primulin)*	Hdac8	CHEMBL2347	Q8VH37	0	0 / 1
Malvidin-3-O-galactoside (Primulin)*	Hdac1	CHEMBL4001	O09106	0	0 / 1
Malvidin-3-O-galactoside (Primulin)*	Map2k1	CHEMBL5860	P31938	0	0 / 1
Malvidin-3-O-galactoside (Primulin)*	Mapk1	CHEMBL2207	P63085	0	0 / 1
Malvidin-3-O-galactoside (Primulin)*	Hif1a	CHEMBL6046	Q61221	0	0 / 15
Malvidin-3-O-galactoside (Primulin)*	Adrb1	CHEMBL3440	P34971	0	1 / 0
Malvidin-3-O-galactoside (Primulin)*	Htr2c	CHEMBL3006	P34968	0	0 / 1
Malvidin-3-O-galactoside (Primulin)*	Rps6ka3	CHEMBL3297641	P18654	0	0 / 10

Malvidin-3-O-galactoside (Primulin)*	Plau	CHEMBL1075311	P06869	0	0 / 2
Malvidin-3-O-galactoside (Primulin)*	S1pr1	CHEMBL1914262	O08530	0	0 / 2
Malvidin-3-O-galactoside (Primulin)*	Abl1	CHEMBL3099	P00520	0	0 / 7
Malvidin-3-O-galactoside (Primulin)*	Ppara	CHEMBL2128	P23204	0	0 / 1
Malvidin-3-O-galactoside (Primulin)*	Il5	CHEMBL1163111	P04401	0	0 / 1
Ferulic acid	Ca15	CHEMBL5973	Q99N23	0.041470299	5 / 3
Ferulic acid	Tlr4	CHEMBL1795167	Q9QUK6	0.041470299	0 / 13
Ferulic acid	Fbp1	CHEMBL5360	Q9QXD6	0.031226558	22 / 0
Ferulic acid	Ace	CHEMBL2994	P09470	0.031226558	16 / 0
Ferulic acid	Ren1	CHEMBL2615	P06281	0.031226558	1 / 0
Ferulic acid	Slc13a5	CHEMBL3769294	Q67BT3	0.031226558	10 / 0
Ferulic acid	Kmo	CHEMBL3407318	Q91WN4	0	18 / 0
Ferulic acid	Ptgdr2	CHEMBL2291	Q9Z2J6	0	5 / 0
Ferulic acid	Mme	CHEMBL2642	Q61391	0	24 / 0
Ferulic acid	Slc6a2	CHEMBL2370	O55192	0	0 / 2
Ferulic acid	Mif	CHEMBL1926491	P34884	0	1 / 1
Ferulic acid	Dao	CHEMBL2331068	P18894	0	8 / 0
Ferulic acid	Ada	CHEMBL3206	P03958	0	6 / 0
Ferulic acid	Ednra	CHEMBL2286	Q61614	0	0 / 22
Ferulic acid	Prkcq	CHEMBL1075295	Q02111	0	0 / 4
Ferulic acid	Hsd17b3	CHEMBL1932905	P70385	0	0 / 1
Ferulic acid	Cda	CHEMBL2110	P56389	0	18 / 0
Ferulic acid	Hcar2	CHEMBL4420	Q9EP66	0	14 / 1
Ferulic acid	Gaa	CHEMBL1667668	P70699	0	3 / 0
Ferulic acid	Slc22a6	CHEMBL5653	Q8VC69	0	2 / 0
Ferulic acid	Ugcg	CHEMBL6013	O88693	0	1 / 0
Ferulic acid	P2rx1	CHEMBL5496	P51576	0	1 / 0
Ferulic acid	Ptger4	CHEMBL2489	P32240	0	0 / 10
Ferulic acid	Gba2	CHEMBL5614	Q69ZF3	0	8 / 0
Ferulic acid	Sell	CHEMBL3162	P18337	0	1 / 0
Ferulic acid	Selp	CHEMBL2455	Q01102	0	1 / 0
Ferulic acid	Sis	CHEMBL1667666	B5THE3	0	3 / 0
Ferulic acid	Anpep	CHEMBL2189140	P97449	0	4 / 0
Ferulic acid	Cdc25b	CHEMBL2723	P30306	0	3 / 0
Ferulic acid	Cacna2d1	CHEMBL4676	O08532	0	9 / 0
Ferulic acid	Gria1	CHEMBL3502	P23818	0	3 / 0
Ferulic acid	Atic	CHEMBL2277	Q9CWJ9	0	1 / 0
Ferulic acid	Dpp4	CHEMBL3883	P28843	0	2 / 0
Ferulic acid	Gba	CHEMBL2278	P17439	0	8 / 0

Ferulic acid	Htr7	CHEMBL4764	P32304	0	1 / 0
Ferulic acid	Clk1	CHEMBL1075280	P22518	0	0 / 3
Ferulic acid	Ahcy	CHEMBL2389	P50247	0	1 / 0
Ferulic acid	Hmgcr	CHEMBL2764	Q01237	0	2 / 1
Ferulic acid	Mapkapk2	CHEMBL4990	P49138	0	1 / 0
Ferulic acid	Casp1	CHEMBL4800	P29452	0	2 / 0
Ferulic acid	Hes1	CHEMBL1075292	P35428	0	1 / 0
Ferulic acid	Upp1	CHEMBL3718	P52624	0	2 / 0
Ferulic acid	Cyp27b1	CHEMBL3329080	O35084	0	0 / 1
Ferulic acid	Ptgdr	CHEMBL3933	P70263	0	0 / 1
Ferulic acid	Ptger3	CHEMBL4336	P30557	0	0 / 3
Ferulic acid	Chrna7	CHEMBL3365	P49582	0	0 / 7
3,4-Dihydroxybenzoic acid (Protocatechuic acid)*	Comt	CHEMBL3286068	O88587	0.043918633	0 / 7
3,4-Dihydroxybenzoic acid (Protocatechuic acid)*	Polb	CHEMBL4565	Q8K409	0	0 / 2
3,4-Dihydroxybenzoic acid (Protocatechuic acid)*	Adrb2	CHEMBL3707	P18762	0	0 / 4
3,4-Dihydroxybenzoic acid (Protocatechuic acid)*	Rarg	CHEMBL4177	P18911	0	0 / 3
3,4-Dihydroxybenzoic acid (Protocatechuic acid)*	Rarb	CHEMBL3266	P22605	0	0 / 3
3,4-Dihydroxybenzoic acid (Protocatechuic acid)*	Rxrg	CHEMBL4402	P28705	0	0 / 1
3,4-Dihydroxybenzoic acid (Protocatechuic acid)*	Rxra	CHEMBL3084	P28700	0	0 / 1
3,4-Dihydroxybenzoic acid (Protocatechuic acid)*	Rara	CHEMBL2792	P11416	0	0 / 2
3,4-Dihydroxybenzoic acid (Protocatechuic acid)*	Adrb3	CHEMBL4030	P25962	0	0 / 2
3,4-Dihydroxybenzoic acid (Protocatechuic acid)*	Hsp90aa1	CHEMBL4197	P07901	0	0 / 2
3,4-Dihydroxybenzoic acid (Protocatechuic acid)*	Trpm8	CHEMBL3108632	Q8R4D5	0	0 / 1
3,4-Dihydroxybenzoic acid (Protocatechuic acid)*	Ces2c	CHEMBL2217	Q91WG0	0	0 / 1
p-Coumaric acid ethyl ester	Trpa1	CHEMBL1075310	Q8BLA8	0	0 / 1
p-Coumaric acid ethyl ester	Ptger2	CHEMBL2488	Q62053	0	0 / 7
p-Coumaric acid ethyl ester	Drd1	CHEMBL3071	Q61616	0	0 / 1
p-Coumaric acid ethyl ester	Slc6a4	CHEMBL4642	Q60857	0	0 / 1
p-Coumaric acid ethyl ester	Oprk1	CHEMBL4329	P33534	0	0 / 1
p-Coumaric acid ethyl ester	Mgll	CHEMBL5774	O35678	0	0 / 1
p-Coumaric acid ethyl ester	Ppard	CHEMBL2458	P35396	0	0 / 2
IsoFerulic acid	Metap2	CHEMBL1075272	O08663	0	1 / 0

IsoFerulic acid	Csnk2a2	CHEMBL5326	O54833	0	2 / 0
IsoFerulic acid	Aoc3	CHEMBL4727	O70423	0	1 / 0
IsoFerulic acid	Notum	CHEMBL3758064	Q8R116	0	2 / 0
IsoFerulic acid	Slc5a2	CHEMBL1075302	Q923I7	0	2 / 0
IsoFerulic acid	Ffar1	CHEMBL5411	Q76JU9	0	1 / 0
Syringic acid	St14	CHEMBL3745587	P56677	0	0 / 2
Syringic acid	Lta4h	CHEMBL3738	P24527	0	2 / 0
Syringic acid	Adam17	CHEMBL4379	Q9Z0F8	0	4 / 0
Syringic acid	Dhfr	CHEMBL4564	P00375	0	0 / 4
Syringic acid	Ptafr	CHEMBL3993	Q62035	0	0 / 2
Syringic acid	Ces1d	CHEMBL3137293	Q8VCT4	0	0 / 1
Syringic acid	Rxrb	CHEMBL4047	P28704	0	0 / 1
Syringic acid	Gcgr	CHEMBL4773	Q61606	0	0 / 3
Syringic acid	Sphk2	CHEMBL1075305	Q9JIA7	0	0 / 2
Syringic acid	Sphk1	CHEMBL2401605	Q8CI15	0	0 / 2
Chlorogenic acid (3-O-Caffeoylquinic acid)	Prkca	CHEMBL2567	P20444	0.097239989	0 / 198
Chlorogenic acid (3-O-Caffeoylquinic acid)	Fpgs	CHEMBL2890	P48760	0	2 / 0
Chlorogenic acid (3-O-Caffeoylquinic acid)	Dnmt1	CHEMBL3351195	P13864	0	4 / 0
Chlorogenic acid (3-O-Caffeoylquinic acid)	Carm1	CHEMBL5538	Q9WVG6	0	1 / 0
Chlorogenic acid (3-O-Caffeoylquinic acid)	Ehmt2	CHEMBL2169718	Q9Z148	0	1 / 0
Chlorogenic acid (3-O-Caffeoylquinic acid)	Mag	CHEMBL1250416	P20917	0	4 / 0
Chlorogenic acid (3-O-Caffeoylquinic acid)	Slc5a1	CHEMBL1744523	Q9QXI6	0	1 / 0
Chlorogenic acid (3-O-Caffeoylquinic acid)	Bcl2a1	CHEMBL1293239	Q07440	0	1 / 0
Chlorogenic acid (3-O-Caffeoylquinic acid)	Gart	CHEMBL3690	Q64737	0	1 / 0
Chlorogenic acid (3-O-Caffeoylquinic acid)	Btk	CHEMBL3259478	P35991	0	1 / 0
Chlorogenic acid (3-O-Caffeoylquinic acid)	Cd22	CHEMBL1075279	P35329	0	1 / 0
Chlorogenic acid (3-O-Caffeoylquinic acid)	Chrm2	CHEMBL3197	Q9ERZ4	0	0 / 11
Sinapic acid	Ptger1	CHEMBL2181	P35375	0	1 / 0
Sinapic acid	Hrh3	CHEMBL3263	P58406	0	1 / 0
Vanillic acid	Ddo	CHEMBL3616357	Q922Z0	0	1 / 0
Vanillic acid	Htr4	CHEMBL2183	P97288	0	0 / 3
Isovanillin	Dusp1	CHEMBL5623	P28563	0	1 / 0

Isovanillin	Nlrp3	CHEMBL3779755	Q8R4B8	0	1 / 0
Isovanillin	Tgm2	CHEMBL2079853	P21981	0	2 / 0
Isovanillin	Ephx1	CHEMBL1075293	Q9D379	0	10 / 0
Isovanillin	Nos3	CHEMBL2643	P70313	0	3 / 0
Isovanillin	Soat1	CHEMBL4464	Q61263	0	1 / 0
Isovanillin	Mapk14	CHEMBL2336	P47811	0	0 / 3
Isovanillin	Pla2g10	CHEMBL4200	Q9QXX3	0	3 / 0
Isovanillin	Faah	CHEMBL3455	O08914	0	1 / 0
Isovanillin	Nat2	CHEMBL5724	P50295	0	1 / 0
Isovanillin	Nampt	CHEMBL3259474	Q99KQ4	0	1 / 0
Isovanillin	Htr5a	CHEMBL3597	P30966	0	4 / 0
Isovanillin	Kcnj2	CHEMBL1293290	P35561	0	0 / 1
Diisobutyl phthalate	Ctsg	CHEMBL5622	P28293	0.100578902	24 / 0
Diisobutyl phthalate	Prep	CHEMBL4935	Q9QUR6	0.100578902	128 / 0
Diisobutyl phthalate	Hcrtr2	CHEMBL2434818	P58308	0.100578902	707 / 0
Diisobutyl phthalate	Hcrtr1	CHEMBL2434819	P58307	0.100578902	572 / 0
Diisobutyl phthalate	Rps6ka2	CHEMBL3351220	Q9WUT3	0.100578902	6 / 0
Diisobutyl phthalate	Mapk11	CHEMBL4335	Q9WUI1	0.100578902	41 / 0
Diisobutyl phthalate	Clk3	CHEMBL1075282	O35492	0.100578902	2 / 0
Diisobutyl phthalate	P2rx7	CHEMBL5183	Q9Z1M0	0.100578902	365 / 0
Diisobutyl phthalate	Gpbar1	CHEMBL1255150	Q80SS6	0.100578902	52 / 0
Diisobutyl phthalate	Hrh4	CHEMBL5657	Q91ZY2	0.100578902	38 / 0
Diisobutyl phthalate	Lyn	CHEMBL2258	P25911	0.100578902	3 / 0
Diisobutyl phthalate	Fgfr4	CHEMBL3839	Q03142	0.100578902	3 / 0
Diisobutyl phthalate	Fgr	CHEMBL2034795	P14234	0.100578902	4 / 0
Diisobutyl phthalate	Tie1	CHEMBL2034800	Q06806	0.100578902	2 / 0
Diisobutyl phthalate	Ros1	CHEMBL2034802	Q78DX7	0.100578902	4 / 0
Diisobutyl phthalate	Trpv4	CHEMBL6126	Q9EPK8	0.100578902	4 / 0
Diisobutyl phthalate	Pde7a	CHEMBL2040702	P70453	0.100578902	58 / 0
Diisobutyl phthalate	Grm1	CHEMBL2892	P97772	0.100578902	47 / 0
Diisobutyl phthalate	Gck	CHEMBL3112387	P52792	0.100578902	78 / 0
Diisobutyl phthalate	Rorc	CHEMBL1293231	P51450	0.100578902	52 / 0
Diisobutyl phthalate	Gpr39	CHEMBL3341584	Q5U431	0.100578902	11 / 0
Diisobutyl phthalate	Shh	CHEMBL5387	Q62226	0	10 / 0
Diisobutyl phthalate	Tdo2	CHEMBL1075307	P48776	0	11 / 0
Diisobutyl phthalate	Scn10a	CHEMBL5158	Q6QIY3	0	14 / 0
Diisobutyl phthalate	Bdkrb1	CHEMBL1250407	Q61125	0	12 / 0
Diisobutyl phthalate	Npy5r	CHEMBL3802	O70342	0	168 / 0
Diisobutyl phthalate	Rapgef4	CHEMBL3593151	Q9EQZ6	0	15 / 0
Diisobutyl phthalate	Ppme1	CHEMBL2189138	Q8BVQ5	0	7 / 0
α -Linolenic Acid*	Scd1	CHEMBL5353	P13516	0.141522086	14 / 1
α -Linolenic Acid*	Ffar4	CHEMBL2052036	Q7TMA4	0.100578902	21 / 0
α -Linolenic Acid*	Npc1l1	CHEMBL1075296	Q6T3U4	0.100578902	0 / 10
α -Linolenic Acid*	Nr3c1	CHEMBL3144	P06537	0.100578902	0 / 2

α -Linolenic Acid*	Hsd11b2	CHEMBL3490	P51661	0.100578902	17 / 19
Linoleic acid	Enpp2	CHEMBL3826871	Q9R1E6	0.100578902	31 / 0
Linoleic acid	Alox5ap	CHEMBL3414408	P30355	0.100578902	96 / 0
Linoleic acid	Nr1i3	CHEMBL3069	O35627	0	0 / 2
Linoleic acid	Mc1r	CHEMBL4077	Q01727	0	1 / 0
linoleic acid	Smo	CHEMBL6080	P56726	0	1 / 0
linoleic acid	Dgat1	CHEMBL1075284	Q9Z2A7	0	107 / 0
linoleic acid	Avpr1a	CHEMBL3414410	Q62463	0	12 / 0
linoleic acid	Cckbr	CHEMBL2854	P56481	0	52 / 0
linoleic acid	Gpam	CHEMBL3580525	Q61586	0	1 / 0
linoleic acid	Dagla	CHEMBL5180	Q6WQJ1	0	5 / 0
linoleic acid	Acacb	CHEMBL3108631	E9Q4Z2	0	1 / 0
linoleic acid	Slc10a2	CHEMBL2073708	P70172	0	10 / 0
linoleic acid	Slc22a1	CHEMBL2073664	O08966	0	1 / 0
1-Linoleoylglycerol	Vdr	CHEMBL5601	P48281	0.097239989	8 / 0
1-Linoleoylglycerol	Scn9a	CHEMBL3414411	Q62205	0.097239989	8 / 0
1-Linoleoylglycerol	Gpr119	CHEMBL5263	Q7TQP3	0.097239989	1 / 0
1-Linoleoylglycerol	Tacr1	CHEMBL2668	P30548	0	4 / 0
1-Linoleoylglycerol	Csf1r	CHEMBL5570	P09581	0	18 / 0
1-Linoleoylglycerol	Lpar4	CHEMBL3321650	Q8BLG2	0	2 / 6
1-Linoleoylglycerol	Lpar1	CHEMBL3621025	P61793	0	2 / 18
1-Linoleoylglycerol	Tbk1	CHEMBL2189160	Q9WUN2	0	11 / 0
1-Linoleoylglycerol	Ccr5	CHEMBL3676	P51682	0	7 / 0
1-Linoleoylglycerol	Inhba	CHEMBL3588734	Q04998	0	1 / 0
1-Linoleoylglycerol	Cxcr3	CHEMBL5200	O88410	0	5 / 0
1-Linoleoylglycerol	Zap70	CHEMBL2034801	P43404	0	1 / 0
Hydroperoxylinoleic acid	Lipg	CHEMBL2380190	Q9WVG5	0	3 / 0
Hydroperoxylinoleic acid	Mogat2	CHEMBL3603729	Q80W94	0	1 / 0
Hydroperoxylinoleic acid	Tbxa2r	CHEMBL1795181	P30987	0	0 / 1
Methyl linolenate	Clk2	CHEMBL1075281	O35491	0.111501865	26 / 0
Methyl linolenate	Clk4	CHEMBL1075283	O35493	0.111501865	59 / 0
Methyl linolenate	Scarb1	CHEMBL1741203	Q61009	0.111501865	36 / 0
Methyl linolenate	Gckr	CHEMBL3232700	Q91X44	0.111501865	11 / 0
Methyl linolenate	Porcn	CHEMBL1255164	Q9JJJ7	0.111501865	34 / 0
Methyl linolenate	Fpr-s1	CHEMBL3407315	O08790	0.111501865	26 / 0
Methyl linolenate	Calcrl	CHEMBL2034811	Q9R1W5	0.111501865	123 / 0
Methyl linolenate	Dck	CHEMBL2570	P43346	0	38 / 0
Methyl linolenate	Gnrhr	CHEMBL3232679	Q01776	0	26 / 0
Elaidic Acid*	Slc22a20	CHEMBL5269	Q80UJ1	0	0 / 5
Elaidic Acid*	Hrh1	CHEMBL4322	P70174	0	5 / 0
Elaidic Acid*	Ntsr1	CHEMBL3570	O88319	0	11 / 0
(7Z)-Hexadecenoic acid	Cckar	CHEMBL2798	O08786	0	17 / 0
Apigenin; 4',5,7-Trihydroxyflavone	Stk3	CHEMBL4310	Q9JI10	0	0 / 1

Apigenin; 4',5,7-Trihydroxyflavone	Acvr1	CHEMBL3309042	P37172	0	0 / 1
Apigenin; 4',5,7-Trihydroxyflavone	Gpr35	CHEMBL2390813	Q9ES90	0	0 / 1
Palmitic acid	Ptgfr	CHEMBL5000	P43117	0.053556076	1 / 1
Palmitic acid	Crabp1	CHEMBL3208	P62965	0.053556076	5 / 0
Palmitic acid	Crabp2	CHEMBL4172	P22935	0.053556076	6 / 0
Palmitic acid	Rnpep	CHEMBL2836	Q8VCT3	0	0 / 2
Hydroxyicosanoic Acid	Pla2g2a	CHEMBL5761	P31482	0	2 / 0
Hydroxyicosanoic Acid	Pla2g5	CHEMBL4167	P97391	0	1 / 0
Hydroxyicosanoic Acid	Pla2g2d	CHEMBL5537	Q9WVF6	0	1 / 0
Hydroxyicosanoic Acid	Acaca	CHEMBL3086	Q5SWU9	0	1 / 0
Hydroxyicosanoic Acid	Grm8	CHEMBL4626	P47743	0	0 / 1
Hydroxyicosanoic Acid	Slc6a11	CHEMBL3699	P31650	0	0 / 1
Hydroxyicosanoic Acid	Slc6a1	CHEMBL5445	P31648	0	0 / 1
Hydroxyicosanoic Acid	Paox	CHEMBL3408	Q8C0L6	0	0 / 1
Hydroxyicosanoic Acid	Slc6a13	CHEMBL5205	P31649	0	0 / 1
Docosanoic acid (Behenic acid)	Ccr3	CHEMBL3406	P51678	0	1 / 0

Table S3. Summary of ALD-Gene Associations. In DisGeNET database, 195 ALD-related genes were obtained.

Disease	UniProt	Gene	DSI_g	DPI_g	Score_gda	First_Ref	Last_Ref
Alcoholic Liver Diseases	Q9NST1	PNPLA3	0.556	0.692	0.4	2010	2019
Alcoholic Liver Diseases	P10451	SPP1	0.353	0.885	0.33	2008	2014
Alcoholic Liver Diseases	P05181	CYP2E1	0.459	0.885	0.3	1997	2018
Alcoholic Liver Diseases	P02753	RBP4	0.493	0.808	0.3	2006	2006
Alcoholic Liver Diseases	Q03181	PPARD	0.513	0.846	0.3	2016	2016
Alcoholic Liver Diseases	P01375	TNF	0.231	0.962	0.3	1995	2016
Alcoholic Liver Diseases	Q30201	HFE	0.436	0.846	0.26	1998	2012
Alcoholic Liver Diseases	P01584	IL1B	0.276	0.962	0.23	2000	2019
Alcoholic Liver Diseases	Q16665	HIF1A	0.327	0.923	0.21	2006	2018
Alcoholic Liver Diseases	P09341	CXCL1	0.449	0.923	0.21	1999	2019
Alcoholic Liver Diseases	Q03135	CAV1	0.388	0.885	0.2	2013	2013
Alcoholic Liver Diseases	P24557	TBXAS1	0.619	0.615	0.2	1997	1997
Alcoholic Liver Diseases	P19875	CXCL2	0.513	0.885	0.2	1999	1999
Alcoholic Liver Diseases	P80162	CXCL6	0.582	0.692	0.2	1999	1999
Alcoholic Liver Diseases	P01344	IGF2	0.39	0.885	0.2	2008	2008
Alcoholic Liver Diseases	Q96H72	SLC39A13	0.666	0.538	0.2	2015	2015
Alcoholic Liver Diseases	P14780	MMP9	0.305	0.923	0.2	2007	2007
Alcoholic Liver Diseases	P13987	CD59	0.469	0.808	0.2	2002	2002
Alcoholic Liver Diseases	P14174	MIF	0.412	0.885	0.2	2001	2001
Alcoholic Liver Diseases	O00487	PSMD14	0.716	0.308	0.2	2009	2009
Alcoholic Liver Diseases	P26842	CD27	0.517	0.692	0.2	2006	2006
Alcoholic Liver Diseases	Q9Y5K5	UCHL5	0.695	0.346	0.2	2009	2009
Alcoholic Liver Diseases	P06858	LPL	0.474	0.808	0.2	2017	2017
Alcoholic Liver Diseases	P00480	OTC	0.565	0.846	0.2	2009	2009
Alcoholic Liver Diseases	P05198	EIF2S1	0.587	0.692	0.2	2010	2010
Alcoholic Liver Diseases	P05091	ALDH2	0.457	0.885	0.09	1988	2004
Alcoholic Liver Diseases	P81172	HAMP	0.433	0.846	0.07	2007	2014
Alcoholic Liver Diseases	O00206	TLR4	0.321	0.962	0.07	2011	2019
Alcoholic Liver Diseases	P08571	CD14	0.392	0.885	0.06	2001	2016
Alcoholic Liver Diseases	P09488	GSTM1	0.38	0.923	0.05	1994	2016
Alcoholic Liver Diseases	P22301	IL10	0.281	0.923	0.05	2000	2016
Alcoholic Liver Diseases	P14550	AKR1A1	0.51	0.846	0.05	1993	2006
Alcoholic Liver Diseases	P00325	ADH1B	0.502	0.846	0.05	1988	2016
Alcoholic Liver Diseases	P05231	IL6	0.248	0.962	0.04	2002	2014
Alcoholic Liver Diseases	Q96EB6	SIRT1	0.378	0.885	0.04	2014	2020
Alcoholic Liver Diseases	P00326	ADH1C	0.547	0.731	0.04	1997	2010
Alcoholic Liver Diseases	Q9BZW4	TM6SF2	0.638	0.462	0.04	2018	2019
Alcoholic Liver Diseases	P40763	STAT3	0.32	0.923	0.03	2003	2014
Alcoholic Liver Diseases	P01137	TGFB1	0.287	0.962	0.03	2002	2008
Alcoholic Liver Diseases	Q07869	PPARA	0.432	0.885	0.03	2008	2018
Alcoholic Liver Diseases	P24298	GPT	0.403	0.923	0.03	2017	2019

Alcoholic Liver Diseases	P01185	AVP	0.437	0.846	0.03	1993	2006
Alcoholic Liver Diseases	P04439	HLA-A	0.37	0.846	0.03	1977	1988
Alcoholic Liver Diseases	O60760	HPGDS	0.388	0.923	0.03	1996	2011
Alcoholic Liver Diseases	P13500	CCL2	0.321	0.962	0.03	1999	2017
Alcoholic Liver Diseases	Q16236	NFE2L2	0.357	0.885	0.03	2014	2018
Alcoholic Liver Diseases	Q9Y572	RIPK3	0.497	0.885	0.02	2013	2014
Alcoholic Liver Diseases	P60709	ACTB	0.325	0.923	0.02	1985	2011
Alcoholic Liver Diseases	P18510	IL1RN	0.373	0.923	0.02	1998	2005
Alcoholic Liver Diseases	Q15848	ADIPOQ	0.376	0.885	0.02	2015	2018
Alcoholic Liver Diseases	P10145	CXCL8	0.31	0.962	0.02	2009	2019
Alcoholic Liver Diseases	Q96RI1	NR1H4	0.513	0.808	0.02	2018	2019
Alcoholic Liver Diseases	P30711	GSTT1	0.393	0.923	0.02	2005	2005
Alcoholic Liver Diseases		MIR21	0.363	0.846	0.02	2014	2018
Alcoholic Liver Diseases	P01583	IL1A	0.333	0.962	0.02	2005	2019
Alcoholic Liver Diseases	P31749	AKT1	0.311	0.962	0.02	2018	2019
Alcoholic Liver Diseases	Q7Z5P4	HSD17B13	0.623	0.538	0.02	2019	2019
Alcoholic Liver Diseases	P21554	CNR1	0.446	0.808	0.02	2017	2019
Alcoholic Liver Diseases	Q06546	GABPA	0.379	0.885	0.02	2018	2018
Alcoholic Liver Diseases	P04179	SOD2	0.379	0.923	0.02	2016	2016
Alcoholic Liver Diseases	P08246	ELANE	0.447	0.846	0.02	2012	2017
Alcoholic Liver Diseases	Q9GZX6	IL22	0.393	0.885	0.02	2013	2018
Alcoholic Liver Diseases	P33897	ABCD1	0.563	0.731	0.02	2000	2013
Alcoholic Liver Diseases	Q92187	ST8SIA4	0.546	0.846	0.02	1997	2004
Alcoholic Liver Diseases	P02771	AFP	0.429	0.885	0.02	2019	2019
Alcoholic Liver Diseases	Q6WCQ1	MPRIP	0.556	0.885	0.02	2013	2014
Alcoholic Liver Diseases	Q8NBJ4	GOLM1	0.56	0.692	0.02	2004	2013
Alcoholic Liver Diseases	P09429	HMGB1	0.368	0.923	0.02	2014	2019
Alcoholic Liver Diseases	P42336	PIK3CA	0.292	0.923	0.01	2018	2018
Alcoholic Liver Diseases	Q13427	PPIG	0.497	0.846	0.01	2004	2004
Alcoholic Liver Diseases	Q99572	P2RX7	0.45	0.808	0.01	2018	2018
Alcoholic Liver Diseases	P54278	PMS2	0.484	0.808	0.01	2014	2014
Alcoholic Liver Diseases	Q9NPH5	NOX4	0.471	0.885	0.01	2017	2017
Alcoholic Liver Diseases	P48736	PIK3CG	0.32	0.885	0.01	2018	2018
Alcoholic Liver Diseases	O60315	ZEB2	0.471	0.808	0.01	2018	2018
Alcoholic Liver Diseases	P54296	MYOM2	0.57	0.692	0.01	2015	2015
Alcoholic Liver Diseases	Q9UBH6	XPR1	0.488	0.731	0.01	2018	2018
Alcoholic Liver Diseases	P37231	PPARG	0.358	0.885	0.01	2017	2017
Alcoholic Liver Diseases	Q9GZP9	DERL2	0.617	0.615	0.01	2019	2019
Alcoholic Liver Diseases	Q7LGC8	CHST3	0.517	0.846	0.01	2008	2008
Alcoholic Liver Diseases	O00329	PIK3CD	0.319	0.885	0.01	2018	2018
Alcoholic Liver Diseases	Q9NRA0	SPHK2	0.546	0.731	0.01	2019	2019
Alcoholic Liver Diseases	P42338	PIK3CB	0.322	0.885	0.01	2018	2018
Alcoholic Liver Diseases	Q9HD89	RETN	0.454	0.808	0.01	2018	2018
Alcoholic Liver Diseases	Q8TC59	PIWIL2	0.612	0.577	0.01	2019	2019

Alcoholic Liver Diseases	P62195	PSMC5	0.686	0.538	0.01	1997	1997
Alcoholic Liver Diseases	P08922	ROS1	0.439	0.885	0.01	2017	2017
Alcoholic Liver Diseases	Q13586	STIM1	0.501	0.808	0.01	2018	2018
Alcoholic Liver Diseases	Q9H9Z2	LIN28A	0.523	0.731	0.01	2017	2017
Alcoholic Liver Diseases		MIR570	0.682	0.346	0.01	2015	2015
Alcoholic Liver Diseases	P01135	TGFA	0.432	0.885	0.01	2003	2003
Alcoholic Liver Diseases	O60603	TLR2	0.361	0.923	0.01	2019	2019
Alcoholic Liver Diseases	Q96N66	MBOAT7	0.612	0.5	0.01	2018	2018
Alcoholic Liver Diseases	P13501	CCL5	0.403	0.885	0.01	2003	2003
Alcoholic Liver Diseases		KRT8P3	0.644	0.538	0.01	2006	2006
Alcoholic Liver Diseases	P09936	UCHL1	0.482	0.885	0.01	2015	2015
Alcoholic Liver Diseases	P55851	UCP2	0.493	0.808	0.01	2017	2017
Alcoholic Liver Diseases	P06132	UROD	0.521	0.769	0.01	1976	1976
Alcoholic Liver Diseases	P17861	XBP1	0.477	0.846	0.01	2009	2009
Alcoholic Liver Diseases	P98155	VLDLR	0.558	0.692	0.01	2014	2014
Alcoholic Liver Diseases	Q96DB2	HDAC11	0.636	0.654	0.01	2017	2017
Alcoholic Liver Diseases	O14990	PPP1R2C	0.478	0.846	0.01	1998	1998
Alcoholic Liver Diseases	O14497	ARID1A	0.455	0.846	0.01	2017	2017
Alcoholic Liver Diseases	P26447	S100A4	0.448	0.769	0.01	2019	2019
Alcoholic Liver Diseases	P0DJI8	SAA1	0.513	0.808	0.01	2015	2015
Alcoholic Liver Diseases	P0DJI9	SAA2	0.568	0.731	0.01	2015	2015
Alcoholic Liver Diseases	Q8TB45	DEPTOR	0.626	0.654	0.01	2018	2018
Alcoholic Liver Diseases	Q13309	SKP2	0.492	0.808	0.01	1997	1997
Alcoholic Liver Diseases	P16581	SELE	0.447	0.808	0.01	2013	2013
Alcoholic Liver Diseases	Q5T4F7	SFRP5	0.559	0.692	0.01	2016	2016
Alcoholic Liver Diseases	O95760	IL33	0.409	0.885	0.01	2019	2019
Alcoholic Liver Diseases	O75469	NR1I2	0.418	0.846	0.01	2018	2018
Alcoholic Liver Diseases	Q6DKJ4	NXN	0.584	0.577	0.01	2018	2018
Alcoholic Liver Diseases	O95342	ABCB11	0.566	0.654	0.01	2012	2012
Alcoholic Liver Diseases	P15907	ST6GAL1	0.569	0.731	0.01	2008	2008
Alcoholic Liver Diseases	P29466	CASP1	0.413	0.885	0.01	1997	1997
Alcoholic Liver Diseases	Q8NER1	TRPV1	0.446	0.885	0.01	2015	2015
Alcoholic Liver Diseases	P11245	NAT2	0.451	0.885	0.01	2002	2002
Alcoholic Liver Diseases	P04798	CYP1A1	0.436	0.846	0.01	1994	1994
Alcoholic Liver Diseases	P12821	ACE	0.328	0.923	0.01	2018	2018
Alcoholic Liver Diseases	P35638	DDIT3	0.467	0.846	0.01	2013	2013
Alcoholic Liver Diseases	P30556	AGTR1	0.423	0.846	0.01	2012	2012
Alcoholic Liver Diseases	P18146	EGR1	0.433	0.923	0.01	2017	2017
Alcoholic Liver Diseases	P07099	EPHX1	0.5	0.846	0.01	2000	2000
Alcoholic Liver Diseases	P31751	AKT2	0.474	0.769	0.01	2019	2019
Alcoholic Liver Diseases	P00352	ALDH1A1	0.46	0.846	0.01	1988	1988
Alcoholic Liver Diseases	P30837	ALDH1B1	0.691	0.462	0.01	1989	1989
Alcoholic Liver Diseases	P15121	AKR1B1	0.491	0.885	0.01	2017	2017
Alcoholic Liver Diseases	Q9NTG7	SIRT3	0.488	0.731	0.01	2019	2019

Alcoholic Liver Diseases	Q15393	SF3B3	0.792	0.231	0.01	2018	2018
Alcoholic Liver Diseases	P16050	ALOX15	0.502	0.846	0.01	2017	2017
Alcoholic Liver Diseases	P42345	MTOR	0.343	0.885	0.01	2017	2017
Alcoholic Liver Diseases	P19526	FUT1	0.541	0.692	0.01	2018	2018
Alcoholic Liver Diseases	Q6ZMG9	CERS6	0.65	0.615	0.01	2018	2018
Alcoholic Liver Diseases	Q8NBP7	PCSK9	0.482	0.808	0.01	2019	2019
Alcoholic Liver Diseases	P16410	CTLA4	0.369	0.923	0.01	2004	2004
Alcoholic Liver Diseases	P29279	CCN2	0.399	0.846	0.01	2011	2011
Alcoholic Liver Diseases	O14594	NCAN	0.67	0.5	0.01	2014	2014
Alcoholic Liver Diseases	Q9Y243	AKT3	0.512	0.808	0.01	2019	2019
Alcoholic Liver Diseases		PSC	0.603	0.731	0.01	2013	2013
Alcoholic Liver Diseases	P43490	NAMPT	0.502	0.808	0.01	2019	2019
Alcoholic Liver Diseases	P42771;Q8N726	CDKN2A	0.3	0.885	0.01	2014	2014
Alcoholic Liver Diseases	P19883	FST	0.513	0.731	0.01	2018	2018
Alcoholic Liver Diseases	P06731	CEACAM5	0.42	0.846	0.01	1983	1983
Alcoholic Liver Diseases	Q96TA2	YME1L1	0.633	0.654	0.01	2018	2018
Alcoholic Liver Diseases	Q96RJ3	TNFRSF13C	0.54	0.731	0.01	2019	2019
Alcoholic Liver Diseases	Q99541	PLIN2	0.564	0.692	0.01	2018	2018
Alcoholic Liver Diseases	P07327	ADH1A	0.647	0.577	0.01	2010	2010
Alcoholic Liver Diseases	P08319	ADH4	0.641	0.462	0.01	2004	2004
Alcoholic Liver Diseases	P11766	ADH5	0.604	0.731	0.01	2004	2004
Alcoholic Liver Diseases	Q6UW15	REG3G	0.716	0.385	0.01	2019	2019
Alcoholic Liver Diseases	P35318	ADM	0.436	0.808	0.01	2018	2018
Alcoholic Liver Diseases	Q86UG4	SLCO6A1	0.412	0.885	0.01	1996	1996
Alcoholic Liver Diseases	Q5JSL3	DOCK11	0.606	0.731	0.01	2018	2018
Alcoholic Liver Diseases	Q16539	MAPK14	0.379	0.923	0.01	2003	2003
Alcoholic Liver Diseases	Q9ULY5	CLEC4E	0.695	0.5	0.01	2018	2018
Alcoholic Liver Diseases	Q9NSA1	FGF21	0.485	0.769	0.01	2017	2017
Alcoholic Liver Diseases	P05783	KRT18	0.53	0.692	0.01	2017	2017
Alcoholic Liver Diseases	P41159	LEP	0.349	0.846	0.01	2018	2018
Alcoholic Liver Diseases	O00327	ARNTL	0.51	0.885	0.01	2018	2018
Alcoholic Liver Diseases		MIR122	0.468	0.808	0.01	2019	2019
Alcoholic Liver Diseases		MIR155	0.384	0.885	0.01	2017	2017
Alcoholic Liver Diseases		MIR200A	0.519	0.808	0.01	2018	2018
Alcoholic Liver Diseases		MIR203A	0.471	0.808	0.01	2018	2018
Alcoholic Liver Diseases		MIR212	0.535	0.808	0.01	2008	2008
Alcoholic Liver Diseases		MIR223	0.433	0.808	0.01	2017	2017
Alcoholic Liver Diseases		MIR30E	0.576	0.769	0.01	2017	2017
Alcoholic Liver Diseases		MIR34A	0.421	0.846	0.01	2012	2012
Alcoholic Liver Diseases	P32121	ARRB2	0.561	0.769	0.01	2018	2018
Alcoholic Liver Diseases	Q00266	MAT1A	0.582	0.577	0.01	2005	2005
Alcoholic Liver Diseases	P30301	MIP	0.507	0.846	0.01	1999	1999

Alcoholic Liver Diseases	P43246	MSH2	0.406	0.808	0.01	2014	2014
Alcoholic Liver Diseases	Q02817	MUC2	0.486	0.808	0.01	2013	2013
Alcoholic Liver Diseases	Q16621	NFE2	0.628	0.577	0.01	1997	1997
Alcoholic Liver Diseases	P05787	KRT8	0.517	0.654	0.01	2006	2006
Alcoholic Liver Diseases	Q9Y2Q3	GSTK1	0.412	0.885	0.01	1996	1996
Alcoholic Liver Diseases	Q14653	IRF3	0.517	0.808	0.01	2014	2014
Alcoholic Liver Diseases	Q9NZH6	IL37	0.459	0.846	0.01	2018	2018
Alcoholic Liver Diseases	O94925	GLS	0.489	0.808	0.01	2007	2007
Alcoholic Liver Diseases	O15217	GSTA4	0.678	0.462	0.01	2018	2018
Alcoholic Liver Diseases	P09211	GSTP1	0.383	0.923	0.01	2011	2011
Alcoholic Liver Diseases	Q9UHD0	IL19	0.558	0.769	0.01	2005	2005
Alcoholic Liver Diseases	P52789	HK2	0.515	0.769	0.01	1998	1998
Alcoholic Liver Diseases	P06340	HLA-DOA	0.509	0.808	0.01	2004	2004
Alcoholic Liver Diseases	P11021	HSPA5	0.434	0.885	0.01	2014	2014
Alcoholic Liver Diseases	P98160	HSPG2	0.438	0.885	0.01	2018	2018
Alcoholic Liver Diseases	P02647	APOA1	0.429	0.885	0.01	2005	2005
Alcoholic Liver Diseases	Q8TDS4	HCAR2	0.631	0.692	0.01	2018	2018
Alcoholic Liver Diseases	Q5NUL3	FFAR4	0.621	0.577	0.01	2017	2017
Alcoholic Liver Diseases	P14778	IL1R1	0.509	0.769	0.01	2018	2018
Alcoholic Liver Diseases	P60568	IL2	0.336	0.885	0.01	2008	2008
Alcoholic Liver Diseases	P40189	IL6ST	0.475	0.808	0.01	2009	2009
Alcoholic Liver Diseases	P29460	IL12B	0.48	0.885	0.01	2018	2018
Alcoholic Liver Diseases	Q14005	IL16	0.501	0.846	0.01	2018	2018
Alcoholic Liver Diseases	P19838	NFKB1	0.396	0.923	0.01	2018	2018
